# Live Imaging of Heart Injury in Larval Zebrafish Reveals a Multi-Stage Model of Neutrophil and Macrophage Migration

**DOI:** 10.3389/fcell.2020.579943

**Published:** 2020-10-19

**Authors:** Aryan Kaveh, Finnius A. Bruton, Charlotte Buckley, Magdalena E. M. Oremek, Carl S. Tucker, John J. Mullins, Jonathan M. Taylor, Adriano G. Rossi, Martin A. Denvir

**Affiliations:** ^1^Centre for Cardiovascular Science, Queen’s Medical Research Institute, University of Edinburgh, Edinburgh, United Kingdom; ^2^Strathclyde Institute of Pharmacy and Biomedical Sciences, University of Strathclyde, Glasgow, United Kingdom; ^3^Centre for Inflammation Research, Queen’s Medical Research Institute, University of Edinburgh, Edinburgh, United Kingdom; ^4^Department of Physics, University of Glasgow, Glasgow, United Kingdom

**Keywords:** zebrafish, heart, tail, injury, neutrophil, macrophage, migration, imaging

## Abstract

Neutrophils and macrophages are crucial effectors and modulators of repair and regeneration following myocardial infarction, but they cannot be easily observed *in vivo* in mammalian models. Hence many studies have utilized larval zebrafish injury models to examine neutrophils and macrophages in their tissue of interest. However, to date the migratory patterns and ontogeny of these recruited cells is unknown. In this study, we address this need by comparing our larval zebrafish model of cardiac injury to the archetypal tail fin injury model. Our *in vivo* imaging allowed comprehensive mapping of neutrophil and macrophage migration from primary hematopoietic sites, to the wound. Early following injury there is an acute phase of neutrophil recruitment that is followed by sustained macrophage recruitment. Both cell types are initially recruited locally and subsequently from distal sites, primarily the caudal hematopoietic tissue (CHT). Once liberated from the CHT, some neutrophils and macrophages enter circulation, but most use abluminal vascular endothelium to crawl through the larva. In both injury models the innate immune response resolves by reverse migration, with very little apoptosis or efferocytosis of neutrophils. Furthermore, our *in vivo* imaging led to the finding of a novel wound responsive *mpeg1*+ neutrophil subset, highlighting previously unrecognized heterogeneity in neutrophils. Our study provides a detailed analysis of the modes of immune cell migration in larval zebrafish, paving the way for future studies examining tissue injury and inflammation.

## Introduction

Myocardial infarction occurs when an atherosclerotic plaque ruptures and occludes a coronary artery, resulting in myocardial cell necrosis and loss of contractile function. Immune cells are subsequently recruited to the infarct where they play important roles during the repair process (Dewald et al., 2005; Nahrendorf et al., 2007; Horckmans et al., 2016). However, if not resolved in a timely fashion, this immune response can be detrimental to repair (Dewald et al., 2005; Ma et al., 2013). Therapeutic modulation of the immune response may ameliorate adverse heart inflammation whilst retaining its benefits (Adamo et al., 2020). Such treatments have been shown to improve clinical outcomes following myocardial infarction, but at the expense of a higher risk of infection (Ridker et al., 2017; Tardif et al., 2019). This indicates a clear need to better understand the local and systemic mobilization of immune cells to cardiac injury.

The zebrafish is an invaluable animal model for the study of cardiac injury, repair and immune cell function (Lai et al., 2018). Unlike mammals, zebrafish hearts are highly regenerative, with adults able to fully regenerate up to a quarter of the myocardium in 60 days (Gonzalez-Rosa et al., 2011). Similarly, larval zebrafish are capable of complete heart regeneration within 2 days (Matrone et al., 2013, 2015). Larval zebrafish offer many unique advantages for live imaging as they are small, transparent and genetically tractable, permitting generation of transgenic lines with cell-specific fluorophores. Transgenic lines for two innate immune cells, the neutrophil and macrophage, allow the migration of both cell types to be examined following wounding (Barros-Becker et al., 2017; Rosowski, 2020). Our laboratory established a larval zebrafish model of cardiac injury that produces an accurate and reproducible laser injury at 3 days post fertilization (Matrone et al., 2013; Taylor et al., 2019). This model allows us to closely interrogate local and systemic neutrophil and macrophage responses *in vivo* following cardiac injury, a feat which is currently not possible in other models. Furthermore, it is not known if the immune cell migration sequence in larval zebrafish is consistent across injury models. By directly comparing the heart laser injury to that in the archetypal tail transection model, we seek to determine a conserved sequence of steps involved in immune cell migration to injury.

In this study we use our refined larval zebrafish laser injury model to examine the mobilization of neutrophils and macrophages to cardiac injury. Using a combination of imaging modalities and transgenic tools, we studied each stage of the immune response, starting with egress from hematopoietic tissue, to arrival at the injured myocardium and subsequent resolution of inflammation. We found the majority of both neutrophils and macrophages are recruited to the heart lesion locally and their numbers later resolved by reverse migration. Neutrophils and macrophages are recruited from distal sites and also mobilize into peripheral blood, using abluminal endothelial surfaces of lymphatic and blood vessels as migration highways. Finally, light sheet fluorescence microscopy (LSFM) timelapse imaging identified a novel wound-responsive neutrophil subset defined as *mpx*:GFP+ *mpeg1*:mCherry+, whose role will require further study. In addition to shedding light on previously unappreciated neutrophil heterogeneity, our work provides a multi-stage model of larval zebrafish immune cell migration following injury.

## Results

### Heart Laser Injury Is Characterized by Localized Cardiomyocyte Cell Death

**FIGURE 1:**
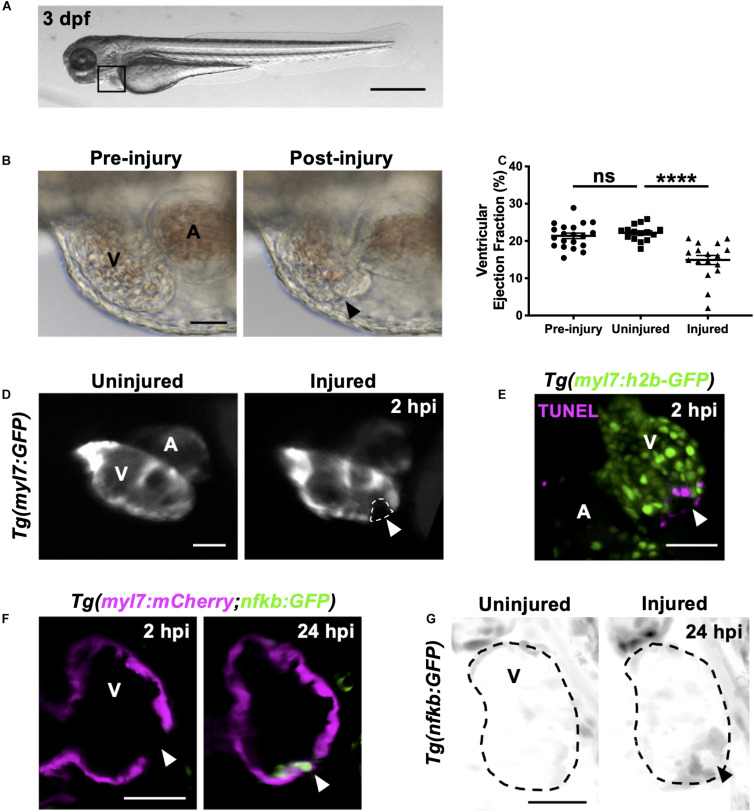
Heart laser injury causes localized cardiomyocyte cell death. **(A)** Whole larva brightfield image of 3 dpf zebrafish with heart region indicated (black square), scale bar = 500 μm. **(B)** Brightfield images of a 3 dpf larval heart pre-laser injury (left) and 1-min post laser injury (right). The lesion is seen as a thickened and partially collapsed region at the ventricular apex (black arrowhead). **(C)** Ventricular ejection fraction before injury, and 2 hpi in injured and uninjured larvae. Error bars = SEM, *n* = 15–20 larvae, experimental *n* = 3. Unpaired *t*-test performed between groups where **** *p* < *0.0001*. **(D)** Epifluorescence images of an uninjured and injured *Tg(myl7:GFP)* heart. Injury site is marked by a loss of myocardial GFP at the ventricular apex (white dashed line and arrowhead). **(E)** 3D LSFM image of a TUNEL stained injured *Tg(myl7:h2b-GFP)* heart at 2 hpi. Injury site is marked by a loss of nuclear myocardial GFP (white arrowhead) bordered by TUNEL positive cells (magenta). Image displayed as a maximum intensity projection (MIP). **(F)** LSFM single *z*-plane image of a *Tg(myl7:mCherry;nfkb:GFP)* ventricle at 2 and 24 hpi following heart injury. White arrowheads indicate loss of myocardial signal at 2 hpi and upregulation of *nfkb:GFP* in wound-bordering cardiomyocytes at 24 hpi. **(G)** 3D LSFM image of *Tg(nfkb:GFP)* ventricular expression at 24 hpi in uninjured and injured larvae (black arrowhead indicates ventricular apex injury site). Image displayed as a MIP (inverse color map). All scale bars = 50 μm unless stated otherwise. V, ventricle; A, atrium; ns, non-significant.

Our laboratory has previously used targeted laser ablation to injure specific regions of the 3 dpf larval zebrafish heart (Figure 1A; Matrone et al., 2013, 2014). We further optimized this approach to induce a localized injury at the ventricular apex of 3 dpf larvae (Supplementary Figure 1-supplement 1). Targeting this region increases reproducibility as it is anatomically unambiguous and minimizes the risk of heart rupture, thus avoiding the release of erythrocytes into the pericardium, which interferes with imaging. These refinements therefore provide a cardiac injury model that more closely mimics a human MI.

Immediately following laser injury, the myocardium at the apex swells and contraction diminishes (Figure 1B). Injured ventricles display a lack of contractility leading to a reduced ventricular ejection fraction compared to uninjured larvae at 2 h post injury (hpi) (Figure 1C and Supplementary Video 1). The injured region is marked by a loss of GFP signal in the cardiomyocyte reporter line *Tg(myl7:GFP)* (Figure 1D and Supplementary Video 1). Staining with propidium iodide (PI) shows this GFP negative region is necrosed myocardium (Supplementary Figure 1-supplement 2A). TUNEL staining of injured hearts at 2 hpi shows the GFP-negative border zone containing apoptotic cardiomyocytes (Figure 1E), which was corroborated using acridine orange staining (Supplementary Figure 1-supplement 2B). To further validate the injury response, we utilized the *Tg(myl7:mCherry;nfkb:GFP)* line to determine if NFkB, an important regulator of programmed cell death, is upregulated following heart injury, as reported in other animal models of MI (Tillmanns et al., 2006; Karra et al., 2015). We observed increased *nfkb* expression in cardiomyocytes bordering the ventricular lesion at 24 hpi (Figure 1F). This ring-like expression pattern (Figure 1G) mimicked TUNEL staining (Figure 1E), again supporting that laser-targeted cardiomyocytes undergo programmed cell death.

### Neutrophils and Macrophages Are Recruited to the Cardiac Injury Site and Display Distinct Recruitment Dynamics

**FIGURE 2:**
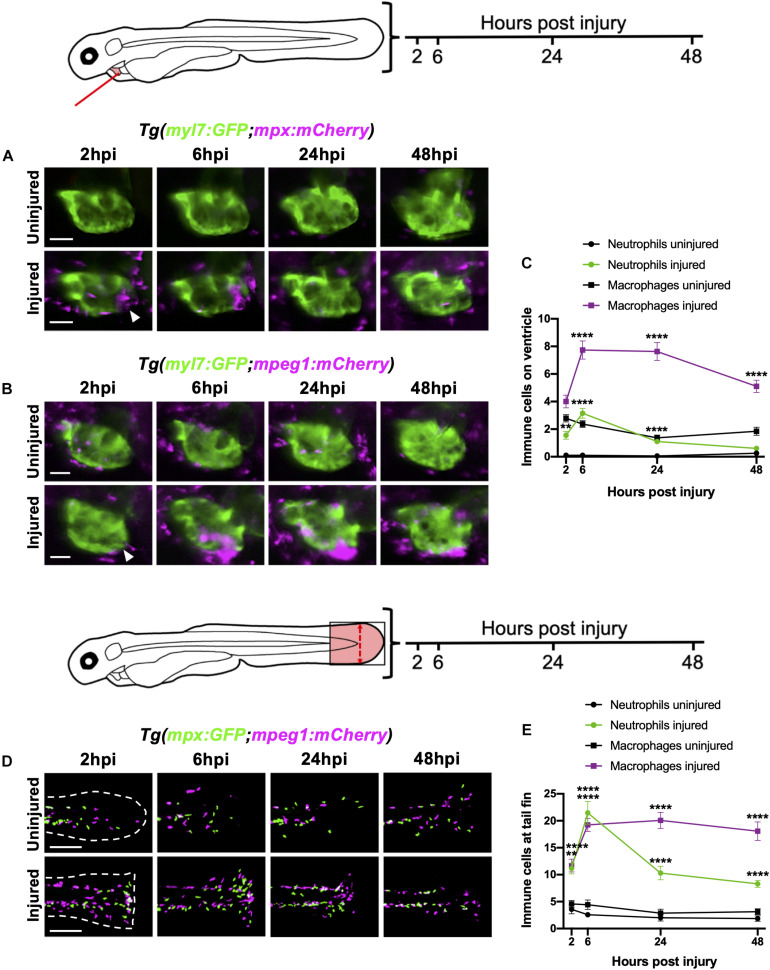
Neutrophils and macrophages display distinct recruitment dynamics during heart injury comparable to tail fin injury. **(A)** Epifluorescence heart images of uninjured and injured *Tg(myl7:GFP;mpx:mCherry)* larvae displaying neutrophil accumulation at 2, 6, 24 and 48 hpi, scale bar = 50 μm. **(B)** Epifluorescence heart images of uninjured and injured *Tg(myl7:GFP;mpeg1:mCherry)* larvae displaying macrophage accumulation at 2, 6, 24 and 48 hpi, scale bar = 50 μm. Arrowheads indicate ventricular apex injury site. **(C)** Neutrophil and macrophage numbers on the ventricle at 2, 6, 24 and 48 hpi for uninjured and injured larvae. Error bars = SEM, *n* = 19 larvae, experimental *n* = 3. **(D)** Epifluorescence tail images of uninjured and injured *Tg(mpx:GFP;mpeg1:mCherry)* larvae displaying neutrophil (green) and macrophage (magenta) accumulation at the timepoints 2, 6, 24 and 48 hpi, scale bar = 60 μm. White dashed line indicates outline of tail fin. **(E)** Neutrophil and macrophage numbers at the tail fin at 2, 6, 24, and 48 hpi for uninjured and injured larvae. Error bars = SEM, *n* = 10 larvae for uninjured groups and *n* = 13 for transected groups, experimental *n* = 3. Two-way ANOVA and Sidak *post hoc* test performed for immune cell comparisons between uninjured and injured groups where ** *p* < *0.001*, **** *p* < *0.0001*. Immune cell counts were made from the region of interest (highlighted red) in the corresponding schematics.

To characterize the recruitment of neutrophils and macrophages to the heart following laser injury we serially imaged *Tg(myl7:GFP;mpx:mCherry)* and *Tg(myl7:GFP;mpeg1:mCherry)* larvae respectively over a two-day period at 2, 6, 24, and 48 hpi using epifluorescence microscopy (Figures 2A,B). Following heart injury, neutrophil numbers on the ventricle increased from 2 hpi, peaked at 6 hpi (3.2 ± 0.4), and gradually resolved to uninjured levels at 48 hpi (0.6 ± 0.2) (Figures 2A,C). While macrophage numbers increased significantly from 6 hpi, cardiac macrophages aggregated at the lesion from 2 hpi (Figure 2B). Macrophage numbers remain elevated at 24 hpi (7.6 ± 0.7), decreasing but not returning to uninjured levels by 48 hpi (5.1 ± 0.4 vs 1.8 ± 0.3) (Figures 2B,C). Both neutrophils and macrophages localized primarily at the ventricular apex injury site (Figures 2A,B and Supplementary Video 2). We also observed neutrophils cyclically migrating around the injured heart, being propelled forwards with each cardiac contraction in real time (Supplementary Video 3).

We contextualized the findings of our heart injury model by comparing it to the well-characterized larval tail fin transection model (Renshaw et al., 2006; Hoodless et al., 2016). Tail fins were transected at 3 dpf and immune cell recruitment quantified at the same timepoints. Similar immune cell dynamics were observed between both models, including a peak neutrophil response at 6 hpi that decreased at 24 hpi and 48 hpi, and a sustained macrophage response during 6–24 hpi, which decreased slightly by 48 hpi (Figures 2D,E). A notable difference between the two injury models was the magnitude of the immune response at 6 hpi, with a 7-fold increase in neutrophils and 2-fold increase in macrophages following tail transection. Additionally, tail inflammation resolved more slowly, with neutrophil numbers remaining relatively high at 48 hpi and macrophage numbers decreasing less steeply between 24 and 48 hpi (Figure 2E).

### Heart Lesion Neutrophils and Macrophages Are Recruited From Both Local and Distal Immune Cell Reservoirs

To determine the origin of neutrophils and macrophages recruited to the heart following injury we used two photoconvertible lines *Tg(mpx:gal4;UAS:kaede)* and *Tg(csf1r:gal4;UAS:kaede)*, which allow spatiotemporal labeling of individual neutrophils and macrophages respectively (Holmes et al., 2012a). By choosing to convert the kaede fluorophore in specific regions of the larva, one can deduce at later timepoints, and in other tissues, whether any immune cells have originated from the converted region. We extended this technique by ‘semi-converting’ regions such that immune cells retain a roughly 1:1 ratio of unconverted and converted kaede, thus allowing assessment of the contribution of three regions simultaneously.

**FIGURE 3:**
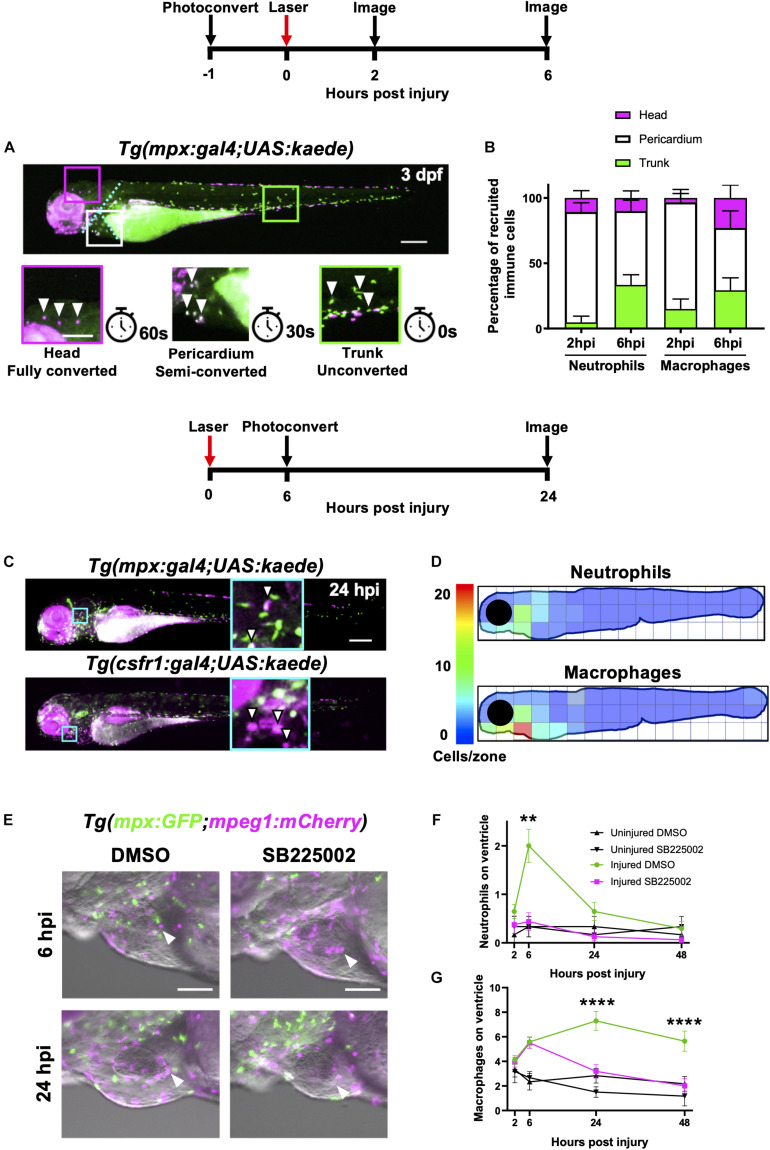
Heart injury recruits local immune cells from the pericardium and head but also cells from distal immune reservoirs. **(A)** Outline of experimental timeline to trace recruited immune cell tissue-origins. Whole body image of a *Tg(mpx:gal4;UAS:kaede)* larva where green cells, unconverted kaede; white cells (magenta and green), semi-converted kaede; and magenta cells, fully converted kaede. The larva is partitioned into three conversion areas marked by the cyan dotted line. Photoconversion of each area was achieved using a mercury lamp. Selected regions (boxes) within the three conversion areas are magnified in the lower panels along with the light exposure time for each level of conversion. White arrowheads, cells from each photoconverted region. Pigment in the eye and peripheries of the trunk autofluorescence magenta but are distinguishable from photoconverted cells because they are immobile. Upper panel scale bar = 200 μm and lower panel scale bar = 100 μm. **(B)** Percentage tissue origin of neutrophils and macrophages recruited to the injured ventricle at 2 and 6 hpi. Error bars = SEM, *n* = 11–21 larvae, experimental *n* = 3. **(C)** Outline of experimental timeline to trace immune cell reverse migration dispersal following heart injury. Whole body image of pericardially photoconverted *Tg(mpx:gal4;UAS:kaede)* (top panel) and *Tg(csf1r:gal4;UAS:kaede)* (bottom panel). Examples of converted cells (white arrow heads) are shown in magnified panels (cyan). Unconverted kaede (green), converted kaede (magenta), arrowheads, examples of converted cells, scale bar = 200 μm. **(D)** Heatmap of a 24 hpi larvae summarizing the dispersal of photoconversion-tracked neutrophils (top panel) and macrophages (bottom panel) following heart injury. Individual zone size = 31000 μm^2^ and mean cell count normalized per zone, *n* = 17. **(E)** Epifluorescence images overlaid on a brightfield image showing neutrophil and macrophage numbers following laser heart injury in *Tg(mpx:GFP;mpeg1:mCherry)* larvae treated with Cxcr1/2 antagonist SB225002 (5 μM) or DMSO (0.1%) vehicle. Arrowheads, ventricular apex injury site. Scale bar = 100 μm. **(F)** Neutrophil numbers on the ventricle following laser injury in *Tg(mpx:GFP;mpeg1:mCherry)* larvae treated with Cxcr1/2 antagonist SB225002 (5 μM) or DMSO (0.1%) vehicle. **(G)** Macrophage numbers on the ventricle following laser injury in *Tg(mpx:GFP;mpeg1:mCherry)* larvae treated with Cxcr1/2 antagonist SB225002 (5 μM) or DMSO (0.1%) vehicle. Two-way ANOVA and Tukey *post hoc* test performed for immune cell comparisons between injured SB225002-treated and injured DMSO vehicle-treated larvae where ** *p* < *0.01* and **** *p* < *0.0001*. Error bars = SEM, *n* = 17 larvae, experimental *n* = 3.

We divided each larva into three anatomical zones (1) head (proximal), (2) pericardium (local) and (3) trunk (distal) which were photoconverted fully, partially or left unconverted respectively. Cells were photoconverted one hour prior to injury and then imaged at 2 hpi and again at the peak immune response at 6 hpi (Figure 3A). At 2 hpi, the majority of both neutrophils and macrophages recruited to the heart were pericardial in origin (84 ± 6.9% and 82 ± 9.8% respectively), with a very small proportion originating from the head (10 ± 5.5% and 3.3 ± 3.3%, respectively) (Figure 3B). The most distal zone (the trunk) contributed very few neutrophils at 2 hpi, however a substantial proportion of heart recruited macrophages originated from the trunk (15 ± 7.6%). Later at 6hpi, we observed an increase in the proportion of trunk-derived neutrophils and macrophages (33 ± 7.7% and 29 ± 9.5%, respectively). Indeed, the absolute number of pericardium-derived immune cells did not increase at 6 hpi, and the overall increase in numbers for each cell type was largely due to the arrival of trunk-derived immune cells (Supplementary Figure 3-supplement 1A). Finally, whilst the proportion of neutrophils originating from the head remained low at 6 hpi, a substantial proportion of the peak macrophage response was head-derived (23 ± 9.9%, Figure 3B).

### Heart Lesion Neutrophils and Macrophages Resolve by Local Dispersal

Wounding studies in larval zebrafish have previously shown that neutrophils primarily undergo reverse migration to resolve their numbers (Mathias et al., 2006; Holmes et al., 2012a,b; Ellett et al., 2015). To test the hypothesis that reverse migration contributes to resolution of immune cell numbers following heart injury, we again utilized photoconvertible lines *Tg(mpx:gal4;UAS:kaede)* and *Tg(csf1r:gal4;UAS:kaede).* In this experiment, immune cells in the pericardial region were photoconverted at the peak response at 6 hpi. Larvae were subsequently re-imaged at 24 hpi to assess the location of any neutrophils or macrophages that may have reverse migrated from the injury (Figure 3C). Summarized by the heatmaps in Figure 3D, neutrophils can be seen to disperse in a posterior and dorsal direction away from the injury. In contrast, macrophages appear to disperse more evenly in all directions. Interestingly, neither cell type moves more than approximately 300 μm from the pericardial region despite our previous analysis demonstrating they can migrate much greater distances over a shorter timeframe during recruitment (Figure 3B). Notably, neither cell type reverse-migrates to any particular organ. Following tail resection, LSFM timelapse imaging suggests that a small number of neutrophils and macrophages undergo cell death at the wound (*n* = 6 timelapses up to 24 hpi) (Supplementary Videos 4, 5). However, only immune cell reverse migration was observed following heart injury (*n* = 12 timelapses up to 24 hpi).

### Cxcr1/2 Is Required for Neutrophil Migration to the Lasered Heart

Following the discovery that both neutrophils and macrophages attend the heart lesion from local and distal sites, we posited that there are chemokine attractants coordinating this response. The best characterized of these chemokines in zebrafish is the Cxcl8-Cxcr1/2 axis, which is required for neutrophil mobility (Deng et al., 2013; Powell et al., 2017; Zuñiga-Traslaviña et al., 2017). We first validated a well-characterized Cxcr1/2 antagonist SB225002 following tail transection and confirmed that it decreased the peak neutrophil response but did not affect macrophage recruitment (Supplementary Figures 3-supplement 1C,D). To determine if Cxcr1/2 is involved in neutrophil or macrophage recruitment to the heart, we performed the same experiment in our heart injury model. The Cxcr1/2 antagonist decreased both neutrophil and macrophage presence at the heart lesion (Figure 3E). The neutrophil response to injury appeared entirely abolished (Figure 3F). Interestingly, macrophage dynamics were unaffected by the drug up until 6 hpi, but significantly decreased at 24 hpi and 48 hpi (Figure 3G). These later timepoints are when macrophages recruited from distal sites would normally reach the heart (Figure 3B), suggesting these immune cells are migrating to the injury site via the action of chemokine attractants.

### Macrophages and Neutrophils Are Mobilized From the Caudal Hematopoietic Tissue Following Heart and Tail Fin Injury

Having established that a significant proportion of late-recruited immune cells are from the distal trunk region, we next tested if heart injury was able to mobilize the cells specifically from the caudal hematopoietic tissue (CHT). The CHT is the predominant source of immune cells in the trunk and is known to contribute to wound neutrophil numbers following tail fin injury (Yoo and Huttenlocher, 2011).

**FIGURE 4:**
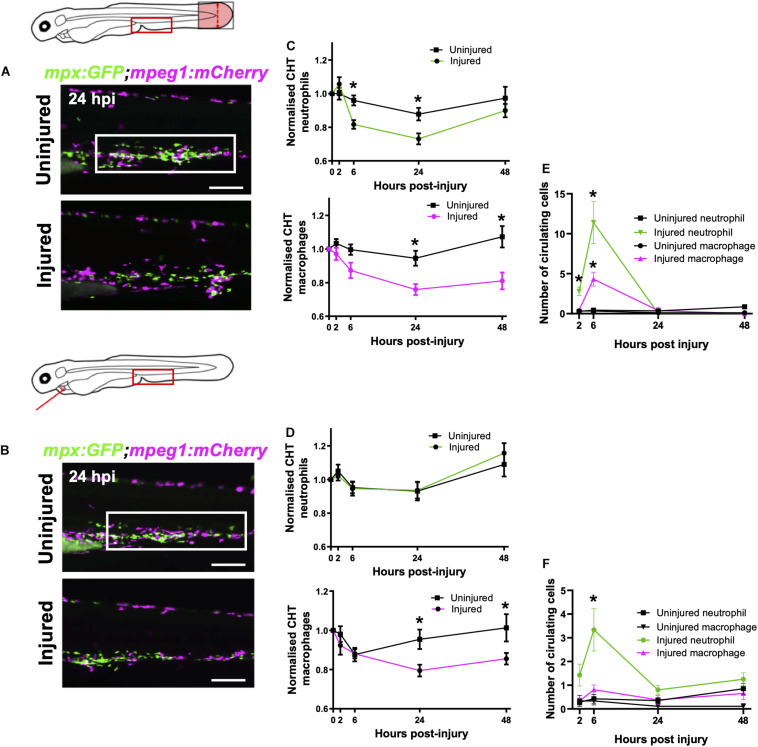
Macrophages are mobilized from the CHT and neutrophils are mobilized into peripheral blood following tail and heart injury. **(A,B)** Epifluorescence images of *Tg(mpx:GFP;mpeg1:mCherry)* larval caudal hematopoietic tissue (CHT) at 24 hpi (macrophages, magenta and neutrophils, green) following tail transection **(A)** and laser heart injury **(B)**. White boxes, area of quantification. Scale bar = 200 μm. **(C,D)** Total CHT neutrophil and macrophage cell area based on normalized fluorescence using *Tg(mpx:GFP;mpeg1:mCherry)* larvae, *n* = 19 larvae, experimental *n* = 3. Values at each timepoint, cell type (neutrophil and macrophage) and injury model (heart laser and tail transection) are indicated. **(E)** Numbers of neutrophils and macrophages in the circulatory system following tail transection, *n* = 16 larvae, experimental *n* = 3. **(F)** Numbers of neutrophils and macrophages in the circulatory system following heart laser injury, *n* = 16 larvae, experimental *n* = 3. For all graphs, error bars, SEM and comparisons between uninjured, transected or laser injured groups was performed by Two-way ANOVA followed by Sidaks multiple comparison test where * p < *0.05*.

First, we sought to verify whether the tail fin injury, which recruits many more immune cells than our heart injury, was capable of inducing CHT emptying of immune cells. Comparison of the CHT in uninjured controls and tail transected *Tg(mpx:GFP;mpeg1:mCherry)* larvae highlighted a reduction in the number of macrophages and neutrophils following injury (Figure 4A). The CHT depleted of neutrophils from 6–24 hpi, replenishing by 48 hpi. Macrophage CHT depletion was detected at 24 hpi and did not recover by 48 hpi (Figure 4C). Unlike tail injury, heart injury did not appear to stimulate a detectable decrease in the presence of CHT neutrophils (Figures 4B,D). However, heart injury caused a decrease in macrophage CHT presence at 24 and 48 hpi (Figures 4B,D), suggesting that macrophages migrate out of the CHT following heart injury.

### Neutrophils Are Mobilized Into Peripheral Blood Following Heart and Tail Fin Injury

In response to cardiac and skin wounds, mammalian neutrophils and monocytes mobilize into the circulatory system in order to rapidly arrive at the site of injury (Anderson and Anderson, 1976). We tested if immune cells circulate in peripheral blood following heart injury, as this has been observed following tail transection (Zuñiga-Traslaviña et al., 2017).

Live epifluorescence imaging allowed us to easily observe and quantify the number of circulating neutrophils and macrophages following either tail or heart injury using the *Tg(mpx:GFP;mpeg1:mCherry)* line. Whilst there were almost no circulating macrophages or neutrophils during uninjured conditions, an increased number of both cell types were detected in circulation following tail transection at 6 hpi (4.3 ± 0.9 and 11.4 ± 2.7, respectively) (Figure 4E). Neutrophils were detected in the blood at 2 and 6 hpi whilst macrophages were only detected at 6 hpi. Similarly, we detected a number of neutrophils in the circulation of heart injured larvae, albeit fewer than in tail transected larvae (3.3 ± 0.9 vs 11.4 ± 2.7) (Figure 4F). In both injury models we observed trans-endothelial migration of neutrophils from the CHT into the cardinal vein, where they became spherical and began to roll or flow freely in the blood stream (Supplementary Video 6). We also observed rolling and egress of macrophages from the CHT following tail transection (Supplementary Video 7). Although we measured a reduction in CHT macrophages following heart injury, and acquired images of macrophages exiting the CHT into the cardinal vein (CV), there was not a statistically significant increase in circulatory macrophages at any time point (Figure 4F).

### Neutrophils and Macrophages Use Vessels as Routes to Tail and Heart Injuries

Having shown that increased macrophage numbers at the heart lesion from 6 hpi were not accompanied by increased circulating macrophages, we next sought to determine if there were other routes and modes of immune cell migration from distal tissues.

**FIGURE 5:**
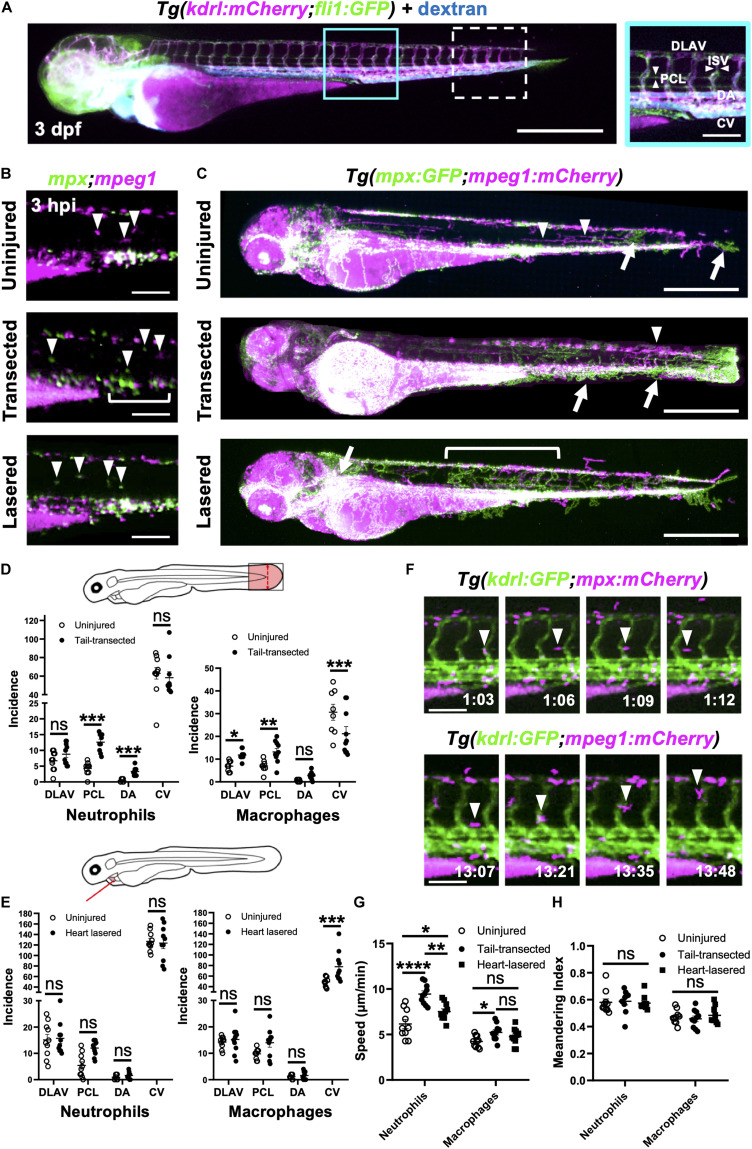
Neutrophils and macrophages utilize blood and lymphatic vessel surfaces for migration following injury. **(A)** Epifluorescence image of a 3dpf *Tg(kdrl:mCherry;fli1:eGFP)* whole larva injected with blue fluorescent 500 kDa dextran highlighting the entire cardiovascular network. Cyan box, region used for quantification for **(D)**, which is magnified in the right panel. White dashed box = region used for quantification for **(E)**. The right panel (**A** magnified) is annotated with vessels monitored for subsequent route use analysis. Left panel scale bar = 500 μm and the right panel scale bar = 100 μm. **(B)** Epifluorescence images of the peri-cloaca trunk region of *Tg(mpx:GFP;mpeg1:mCherry)* larvae at 3 hpi in uninjured, tail transected and heart lasered larvae. Neutrophils, green and macrophages, magenta. White bracket indicates a partially emptied CHT and white arrowheads CHT-liberated neutrophils and macrophages. Scale bar = 100 μm. **(C)** Epifluorescence images superimposed over 4–8 hpi to generate neutrophil (green) and macrophage (magenta) pseudotracks in whole *Tg(mpx:GFP;mpeg1:mCherry)* uninjured, tail transected and heart lasered larvae. White arrowheads highlight representative macrophage tracks, white arrows highlight representative neutrophil tracks and the white bracket highlights a region of neutrophil ventro-dorsal migration. Scale bar = 500 μm. **(D,E)** Incidence of vessel use by neutrophils or macrophages in larvae between 1–12 hpi following tail transection **(D)** or laser heart injury **(E)**. Injured groups were compared to uninjured groups using multiple *t*-tests with subsequent FDR correction using two stage step-up of Benjamini, Kreiger and Yekutieli, where *p < 0.05, **p < 0.01, and *** *p* < *0.001*, *n* = 10 larvae analyzed per group. **(F)** Epifluorescence image sequence from heart lasered larvae showing a neutrophil migrating along the PCL (top) and a macrophage migrating up an ISV (bottom). Hours post injury as indicated. Scale bar = 100 μm. Neutrophil and macrophage speed **(G)** and meandering index **(H)** across the trunk 1.5–8 hpi in tail transected, heart lasered or uninjured larvae. Comparisons between groups were carried out using one-way ANOVA followed by Sidak-Holm multiple comparison test where * p < *0.05*, ** *p* < *0.01* and **** *p* < *0.0001*, *n* = 10 larvae analyzed per group. Error bars for all graphs, SEM; Ns, non-significant; DLAV, dorsal lateral anastomotic vessel; PCL, parachordal lymphatic; DA, dorsal aorta; ISV, intersegmental vessels; and CV, cardinal vein.

Likely candidates were lymphatic vessels and peri-vascular surfaces, as these are known to be rich in adherent proteins and represent a ready-made transport network through the trunk musculature of the larva (von Andrian et al., 1993). To image both vascular and lymphatic networks in the same larva, we crossed two endothelial reporter lines to give *Tg(fli1:eGFP;kdrl:mCherry)* and injected high molecular weight blue fluorescent dextran to visualize any unmarked vessels and fluid spaces (Figure 5A). The vessels, known to run anteriorly posteriorly and ventrally dorsally, were indeed marked by all three of these labels. The anterior-posterior orientated vessels are the dorsal lateral anastomotic vessel (DLAV), dorsal aorta (DA) and CV, whilst the only ventro-dorsal vessels are the intersegmental vessels (ISV) that pattern the length of the larva (Isogai et al., 2001). Additionally, there are parachordal lymphatics (PCL) positioned parallel to the larval notochord (Figure 5A), as reported previously (Jung et al., 2017).

We injected high molecular weight dextran into *Tg(mpx:GFP;mpeg1:mCherry)* larvae to visualize all vessels and performed whole larva epifluorescence timelapse imaging. This facilitated simultaneous observation of all neutrophils and macrophages and directly confirmed their migration patterns through the trunk musculature for both injury models (Supplementary Video 8). In steady state, neutrophil presence outside the CHT was rarely observed and only macrophages were seen patrolling the musculature (Figure 5B). Temporal maximum intensity projections derived from superimposed epifluorescence images in uninjured larvae show neutrophils and macrophages largely restricted to the CHT and head regions (Figure 5C).

Tail fin transection resulted in marked mobilization of macrophages and neutrophils from the CHT (Figure 5C). While the CHT of uninjured larvae appears as an intense white region of macrophage and neutrophil co-localization, transected larvae have neutrophils liberated dorsally and ventrally and migrating toward the tail transection. Quantification of vessel usage show that both cell types predominantly use the CV for migration in both uninjured and tail transection settings (Figure 5D and Supplementary Figure 5-supplement 1B). Neutrophils increase their use of PCL and the DA following injury to reach the tail wound. In contrast, macrophages take a more dorsal route, increasing their use of the PCL and the DLAV (Figures 5C,D and Supplementary Video 8).

Following heart injury, we observed more anterior-directed immune cell migration than in tail transected larvae (Figure 5C). In contrast to tail transection, macrophages begin to increase their use of the CV, taking a ventral route to the heart, occasionally using other vessels and the yolk sac (Figures 5C,E). Interestingly, neutrophils did not significantly increase their use of anterior-posterior oriented vessels in our quantification area (Figure 5E and Supplementary Figure 5-supplement 1B). Analysis of pseudotracks revealed this is because, following egress from the anterior pole of the CHT, neutrophils move dorsally via ISVs. It is only after that we observed neutrophils moving anteriorly via the DLAV and then through the perinephric region to the heart (Figure 5C and Supplementary Video 8). Close inspection of timelapses revealed that both macrophages and neutrophils migrate along the abluminal surface of vessels rather than inside them (Figure 5F).

In addition to vessel usage, we tested if other aspects of immune cell motility changed following injury, namely meandering and speed. Although macrophage speed only increased after tail transection, neutrophils significantly increase their speed following tail transection and to a lesser extent following heart injury (Figure 5G and Supplementary Figure 5-supplement 1A). Meandering index (MI) was unchanged for both cell types and in both injury settings (Figure 5H), indicating the increase in neutrophil speed is not an artifact of directionality. Taken together our data confirm that immune cells indeed use vessels as routes of migration and that there are unique patterns of use for each cell type and injury setting.

### Neutrophils and Macrophages Migrate Onto the Heart via the Pericardium and Adopt Specific Migratory Behaviors Once at the Injury Site

**FIGURE 6:**
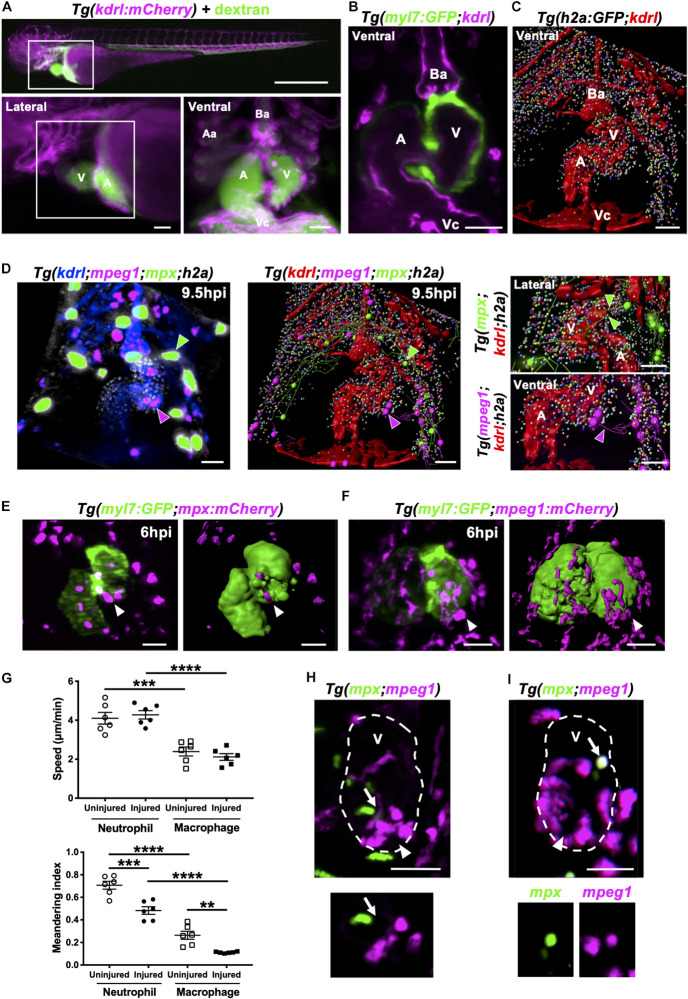
Neutrophils and macrophages migrate onto the ventricle via the pericardium and adopt specific migratory behaviors once on the ventricle following injury. **(A)** Epifluorescence image of *Tg(kdrl:mCherry)* larva injected with blue fluorescent 500 kDa dextran (colored green) to analyze the surrounding pericardium for vasculature (white box). Scale bar = 500 μm (top). Magnified epifluorescence image of the surrounding pericardium where white box indicates the nearby heart region (lower left). Magnified 3D LSFM image displaying the hearts vasculature (magenta) containing fluorescent dextran (green) (lower right). **(B)** LSFM *z*-plane of *Tg(myl7:GFP;kdrl:mCherry)* larva displaying the hearts myocardium and endothelium. **(C)** Surface rendered LSFM *z*-stack of *Tg(h2a:GFP;kdrl:mCherry)* larva displaying the hearts vasculature and surrounding pericardium. **(D)** LSFM fluorescence z-stack differentially color-labeled (left) and surface rendered z-stack (middle and right) of *Tg(kdrl:mCherry;mpeg1:mCherry;mpx:GFP;h2a:GFP)* larva displaying the heart vasculature (blue or red), pericardium (gray or multi-colored), neutrophils (green) and macrophages (magenta) at 9.5 hpi. Magnified view of neutrophil (right top) and macrophage (right bottom) tracks onto the ventricle. Green and magenta arrowheads indicate neutrophils and macrophages tracked to the ventricle respectively for 30 min from 9.5 hpi. **(E)** LSFM image (left) and surface render (right) of a *Tg(myl7:GFP;mpx:mCherry)* injured heart and recruited neutrophils at 6 hpi. **(F)** LSFM image (left) and surface render (right) of a *Tg(myl7:GFP;mpeg1:mCherry)* injured heart and recruited macrophages at 6 hpi. White arrowheads indicate recruited immune cells on the lesioned myocardium. **(G)** Ventricular recruited neutrophil and macrophage speed (top) and meandering index (distance traveled/displacement) (bottom) in heart lasered and uninjured larvae. Average cell behaviors are plotted per larva, *n* = 5 cells tracked per larva and *n* = 6 larvae per group. Error bars, SEM. One-way ANOVA and Tukey *post hoc* test performed for comparisons between mean behavioral values for each larva where ** p < *0.01*, *** p < *0.001*, and ****p < 0.0001. **(H)** LSFM image of a *Tg(mpx:GFP;mpeg1:mCherry)* injured ventricle displaying neutrophils and macrophages at 10 hpi. Arrow indicates a neutrophil and macrophage near the wound (top). LSFM *z*-plane image of the indicated neutrophil and macrophage. Arrow indicated at the same position (bottom). **(I)** LSFM image of a *Tg(mpx:GFP;mpeg1:mCherry)* injured ventricle displaying neutrophils and macrophages at 18 hpi. Arrow indicates a cell co-expressing *mpx:GFP* and *mpeg:mCherry* on the wounded ventricle (top and bottom). Arrowheads indicate the injury site. Outline of ventricle is indicated with a dashed line. All fluorescence images were acquired in 3D using LSFM and displayed as MIPs unless stated otherwise. All scale bar = 50 μm, unless stated otherwise. Ba, bulbus arteriosus; Aa, aortic arches; V, ventricle; A, atrium; Vc, venous cavernous.

Having shown that most neutrophils and macrophages are recruited to the heart locally (Figures 3A,B), we next wanted to identify how these cells migrate to the ventricle at a local scale. We used our optical gating system coupled with LSFM to acquire computationally frozen high-resolution 3D images of the beating heart (Taylor et al., 2011, 2012, 2019). We injected *Tg(kdrl:mCherry)* larvae intravenously with fluorescent dextran and applied our heartbeat-synchronized imaging to search for existing lymphatic or coronary vessels that could play a role in guiding immune cells to the injury site. We did not find the heart to have any supporting lymphatics or coronary vessels, only a thin layer of endocardium (Figures 6A,B). We next utilized the pan-chromatin *Tg(h2a:GFP)* line which labels all nuclei to visualize the whole pericardium. Analysis of 3D fluorescence image stacks by surface rendering confirmed no other cellular structures or routes between the pericardium and heart (Figure 6C).

We next performed heartbeat-synchronized imaging in injured *Tg(kdrl:mCherry;mpeg1:mCherry;mpx:GFP;h2a:GFP)* larvae, allowing neutrophil and macrophage chemotaxis to be tracked in 3D over several hours (Taylor et al., 2019). Timelapse imaging revealed that both cell types patrol the pericardium, with the majority of tracks concentrated around the bulbous arteriosus (Figure 6D and Supplementary Video 9). Tracking analysis also revealed that neutrophils and macrophages migrate to the injury site via adjacent points on the pericardial wall, typically on the ventricular side (Figure 6D and Supplementary Video 9).

To improve our understanding of neutrophil and macrophage behavior specifically at the heart lesion, we acquired 3D timelapse videos of *Tg(myl7:GFP;mpx:mCherry)* (Figure 6E) and *Tg(myl7:GFP;mpeg1:mCherry)* (Figure 6F) larvae up to 24 hpi. These timelapse videos demonstrated neutrophil wound swarming and macrophage accumulation at the wound, followed by reverse migration of both cell types (Supplementary Videos 10, 11). Quantification of individual cell behavior revealed that neutrophils migrated faster on the ventricle compared to macrophages following injury (4.3 ± 0.2 μm/min vs 2.1 ± 0.2 μm/min) and in uninjured larvae (4.1 ± 0.3 μm/min vs 2.4 ± 0.2 μm/min) (Figure 6G). However, neutrophil and macrophage speed following injury was no different to uninjured larvae (Figure 6G). Both neutrophils and macrophages reside on the ventricle for significantly longer periods of time following injury (Supplementary Figure 6-supplement 1). The duration of individual macrophage residency on the ventricle is 3-fold higher than neutrophils following injury (545 ± 28 min vs 178 ± 37 min) (Supplementary Figure 6-supplement 1). The speed and duration in part explain the turnover of immune cells on the ventricle and therefore contribute to the overall recruitment dynamics following injury (Figure 2). Furthermore, neutrophils and macrophages recruited to the ventricle displayed greater meandering behavior following injury compared to uninjured larvae (0.48 ± 0.03 MI vs 0.71 ± 0.04 MI and 0.11 ± 0.003 MI vs 0.26 ± 0.04 MI). Additionally, macrophages were more meandering than neutrophils following injury (0.11 ± 0.003 MI vs 0.48 ± 0.03 MI) (Figure 6G). Taken together, these data demonstrate that both immune cells have distinct behavioral characteristics which become altered following heart injury.

### Heartbeat-Synchronized LSFM Imaging of Neutrophils and Macrophages Following Injury Reveals an *mpx*:GFP+ *mpeg1*:mCherry+ Cell Population

We next tested if there were neutrophil-macrophage interactions on the heart following injury, such as macrophage efferocytosis of neutrophils as previously observed in other injury models (Meszaros et al., 1999; Ellett et al., 2011). LSFM timelapse experiments (*n* = 6 up to 24 hpi) using *Tg(mpx:GFP;mpeg1:mCherry)* larvae did not show any notable neutrophil-macrophage interactions on the heart (Figure 6H and Supplementary Video 12). Interestingly, we identified immune cells that were co-expressing the transgenes *mpx:*GFP and *mpeg1:*mCherry (Figure 6I). This observation prompted us to investigate these co-positive cells further as neutrophils are generally thought to be a homogenous cell type.

### *mpx*:GFP+ *mpeg1*:mCherry+ Cells Are Neutrophils and Not Macrophages

**FIGURE 7:**
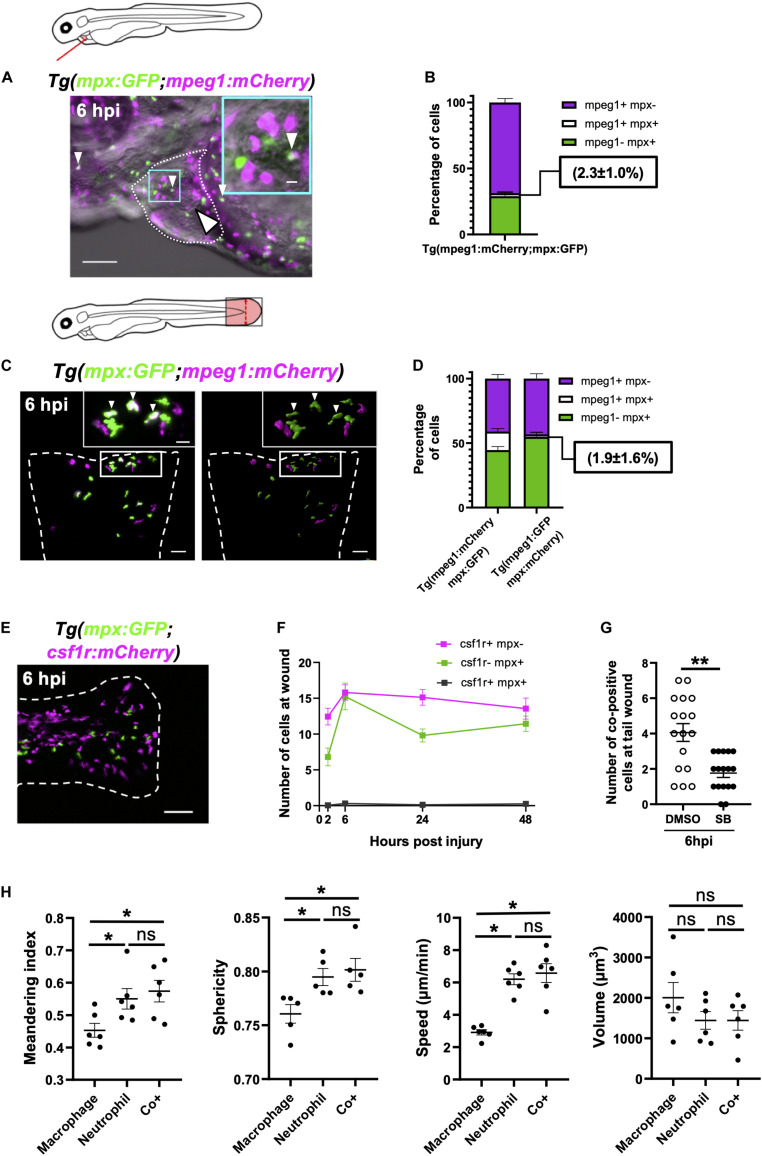
Immune cells co-positive for *mpeg1* and *mpx* expression are neutrophils not macrophages. **(A)** Epifluorescence timelapse still overlaid on a brightfield image displaying a heart lasered *Tg(mpx:GFP;mpeg1:mCherry)* larva at 6 hpi with neutrophils (green), macrophages (magenta) and co-positive cells (white). White dotted line, pericardial region; cyan square, magnified region; small arrowheads, co-positive cells; and the large arrowhead, injury site. Scale bar = 100 μm. **(B)** Percentage of *mpeg1*:mCherry+ *mpx*:GFP-, *mpeg1*:mCherry+ *mpx*:GFP+ and *mpeg1*:mCherry-*mpx*:GFP+ cell populations on the injured ventricle at 6 hpi, *n* = 31 larvae, experimental *n* = 3. **(C)** LSFM 3D images of a transected tail fin at 6 hpi from a *Tg(mpx:GFP;mpeg1:mCherry)* larva displayed as a MIP (left) and surface render (right) where *mpx*:GFP signal is colored green and *mpeg1*:mCherry signal in magenta. Arrowheads, co-positive cells; dashed line, wound edge; and white boxes, magnified panels. Scale bar = 50 μm for main panel and 20 μm for the magnified panel. **(D)** Percentage of *mpeg1*:mCherry+ *mpx*: GFP-, *mpeg1*:mCherry+ *mpx*:GFP+ and *mpeg1*:mCherry-*mpx*:GFP+ cell populations at the tail wound of *Tg(mpx:GFP;mpeg1:mCherry)* and *Tg(mpx:mCherry;mpeg1:GFP)* larvae at 6 hpi, *n* = 24 larvae, experimental *n* = 3. **(E)** Epifluorescence image of a transected tail fin at 6 hpi from a *Tg(mpx:GFP;csf1r:gal4;UAS:mCherry-NTR)* [abbreviated to *Tg(mpx:GFP;csf1r:mCherry*)] larva, showing a lack of co-expressing cells. Scale bar = 100 μm. **(F)** Number of *csf1r*:NTR-mCherry+ *mpx*: GFP-, *csf1r*:NTR-mCherry-*mpx*:GFP+ and *csf1r*:NTR-mCherry+ *mpx*:GFP+ cells at the tail transection wound at 2, 6, 24, and 48 hpi, *n* = 16 larvae, experimental *n* = 3. **(G)** Number of *mpx*:GFP+ *mpeg1*:mCherry+ co-positive cells at the tail transection wound at 6 hpi following treatment with Cxcr1/2 antagonist SB225002 (5 μM) or DMSO (0.1%) vehicle, *n* = 17 larvae, experimental *n* = 3. Comparison between treatments was conducted using a *t*-test where ** p < *0.01*. **(H)** Graphs comparing macrophages, neutrophils and co-positive cells on the basis of meandering index, sphericity, speed and volume derived from 3D analysis of LSFM timelapses from transected *Tg(mpx:GFP;mpeg1:mCherry)* tail fins between 1–2 hpi. Comparisons between cell types was performed by One-way ANOVA followed by a *post hoc* two-stage step-up method of Benjamini, Krieger and Yekutieli FDR correction, *n* = 6 larvae analyzed per group where * p < *0.05*, ns, non-significant. Error bars, SEM for all graphs.

We observed cells expressing both *mpx:*GFP and *mpeg1:*mCherry at and around the injured heart using both LSFM timelapse and epifluorescence microscopy (Figure 7A). This subset of cells represents 2.3 ± 1.0% of *mpx*:GFP+ or *mpeg1*:mCherry+ cells on the injured ventricle (Figure 7B). Co-positive cells are observed more frequently at tail transection wounds (Figure 7C and Supplementary Figure 7-supplement 1A), where they make up a much higher proportion of cells 14.4 ± 2.2% (Figure 7D). Hence, herein we chose the tail transection model to characterize the nature of these cells.

An important question is whether these cells are neutrophils or macrophages We first found indirect evidence that co-positive cells might be neutrophils through comparison with a similar reporter line with different versions of the same transgene *Tg(mpx:mCherry;mpeg1:GFP).* Using this combination of transgenes, we observed very low numbers of wound-associated co-positive cells (1.9 ± 1.6%) (Figure 7D). Comparisons of the proportions of each cell type between these transgenic lines showed that whilst macrophage proportions were very similar between lines, the co-positive cell population observed in *Tg(mpx:GFP;mpeg1:mcherry)* arises at the expense of its neutrophil population. Likewise, it follows that some *mpeg1*-*mpx*+ cells observed in *Tg(mpx:mCherry;mpeg1:GFP)*, might be this same population of co-positive cells, albeit unmarked in this line.

Next, we assessed if co-positive cells were *csf1r*+, a gene not expressed in neutrophils and required for macrophage development (Sweet and Hume, 2003; Chitu and Stanley, 2006). Using *Tg(csf1r:gal4;UAS:mCherry-NTR;mpx:GFP)* larvae, we showed no co-expression of *mpx*:GFP and *csf1r*:mCherry-NTR in recruited cells following tail transection (Figures 7E,F). We can therefore deduce that co-positive cells also do not express *csf1r*, again suggesting that co-positive cells are more neutrophil-like in their fluorophore markers. Finally, we utilized our finding that the pharmacological Cxcr1/2 antagonist (SB225002) inhibits the recruitment of neutrophils but not macrophages following tail transection specifically (Supplementary Figures 3-supplement 1C,D). We reasoned that if co-positive cells were neutrophils then SB225002 should decrease their number at the wound but not if they are macrophages. Tail transected larvae treated with SB225002 did indeed exhibit decreased numbers of co-positive recruited cells at the wound, again suggesting that the co-positive cells are likely neutrophils (Figure 7G and Supplementary Figure 7-supplement 1B).

### Co-positive Immune Cells Represent a Neutrophil Subset

In order to be confident that co-positive cells represent a subset of neutrophils, we excluded the most likely alternative explanations of their *mpx*:GFP+ *mpeg1*:mCherry+ expression. One such explanation is that co-positive cells represent neutrophils that have ingested *mpeg1*:mCherry+ macrophage cellular material. However, analysis of LSFM-acquired images at subcellular resolution refuted this hypothesis. We found co-positive cells to have a uniform intensity distribution of both fluorophores throughout the cytosol and found no phagosomes, suggesting endogenous expression (Supplementary Figure 7-supplement 1C). Next we tested if co-positive cells could represent activated or else developmentally immature neutrophils. Scans of different regions of 3 dpf larvae found these cells in non-hematopoietic tissues such as the perinephric region and brain in addition to the CHT (Supplementary Figure 7-supplement 1D). Furthermore, flow cytometry analysis of *mpx*:GFP+ and *mpeg1*:mCherry+ positive cells isolated from transected tailfins demonstrated that both *mpx*:GFP+ *mpeg1*:mCherry- and co-positive neutrophils degranulate following injury as evidenced by a trend for their side scatter to decrease (Supplementary Figure 7-supplement 2C). Taken together with the fact that co-positive cells attend wounds, it seems unlikely these cells are immature neutrophils. Furthermore, careful analysis of LSFM timelapse videos showed no instances of *mpx*:GFP+ *mpeg1*:mCherry- neutrophils transitioning to co-positive cells (Supplementary Video 13), nor was there any difference in forward scatter between cell types (Supplementary Figure 7-supplement 2B), suggesting that one is not simply an activated form of the other.

Having excluded likely alternative explanations, we proceeded to investigate differences in phenotype between single-positive neutrophils, macrophages and co-positive cells, and whether they might have a specialized role during inflammation. Our flow cytometry analysis did not show any difference in size (FSC) or granularity (SSC) between single-positive or co-positive neutrophils in steady state or injury setting (Supplementary Figures 7-supplement 2A–C). Hence, we took advantage of our unique ability to analyze the behavior of cells *in vivo* to assess if there were obvious behavioral or physical differences between single positive neutrophils, macrophages and co-positive cells. By tracking all three cell types in 3D timelapse to the transected tail fin, we were able to precisely measure speed, sphericity, meandering index and volume (Supplementary Figures 7-supplement 3A–D). No differences were found between co-positive cells and single-positive neutrophils for any of the measures (Figure 7H). However, several differences between macrophages and the other two cell types were found, again indicating that co-positive cells are neutrophils and not macrophages. Specifically, macrophages were found to be more meandering, less spherical, slower and tended to be larger than the other two cell types (Figure 7H). Though our data does not show any obvious structural or behavioral differences between single-positive and co-positive neutrophils, their unique molecular signature and presence at the wound indicate that they are playing a role in wound healing that is yet to be elucidated.

## Discussion

**FIGURE 8:**
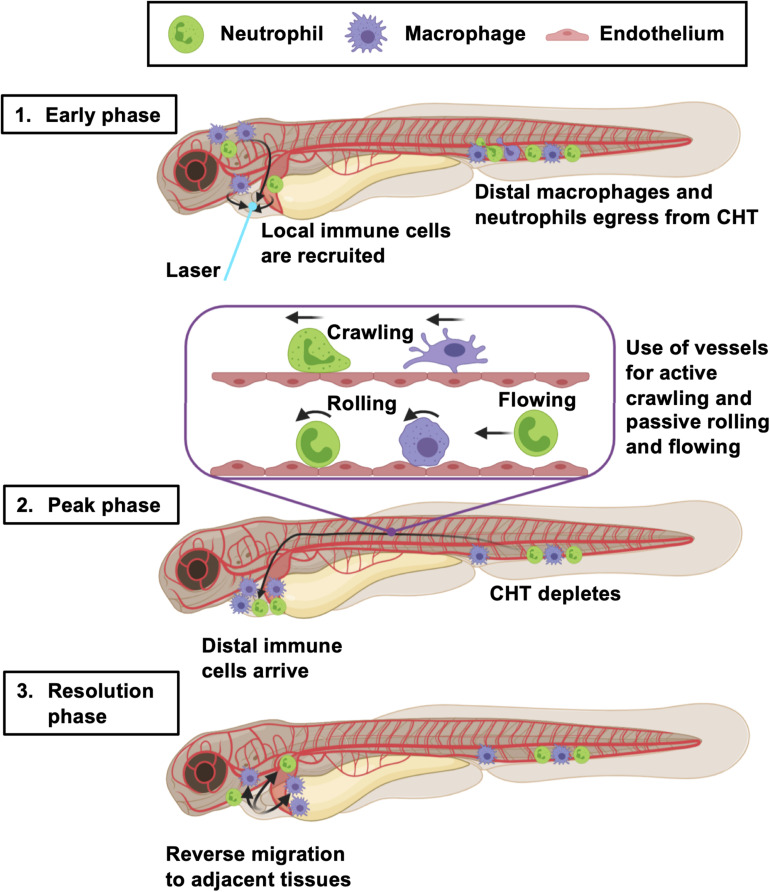
Summary of the larval zebrafish immune response to heart injury. Following laser heart injury in the early phase (0–2 hpi), immune cells resident to nearby pericardial tissues are recruited to the ventricle. At the same time, distal neutrophils and macrophages begin to egress from the CHT into the vascular system. Distal macrophages and neutrophils subsequently utilize blood and lymphatic vessels to migrate to the injured heart, joining locally recruited immune cells during the peak phase (6–24 hpi). The majority of these cells actively crawl along the abluminal surface of vessels, but some also roll and flow along the inside of vessels. The resolution phase is characterized by reverse migration of immune cells to adjacent tissues (> 6 hpi). Created with Biorender.com.

In this study, we provide a novel and in-depth characterization of innate immune cell migration in response to larval zebrafish heart injury. By simultaneously assessing multiple aspects of immune cell mobilization and comparing findings between tail transection and laser heart injury models, we have determined a potentially unifying multi-stage model of the wound immune response in zebrafish larvae (Figure 8).

We suggest that, as in mammalian tissue injury, there is an initial, early peak of neutrophil recruitment followed by a slower accumulation of macrophages. This is consistent with the adult zebrafish tail transection model (Petrie et al., 2014) and has also been observed in many others including larval spinal cord (Tsarouchas et al., 2018), larval trunk vasculature (Gurevich et al., 2018) and adult zebrafish heart injury (Bevan et al., 2019). Interestingly, the relative amplitude of the neutrophil and macrophage response differs between models, as does the duration of response. Whilst the duration can likely be attributed to injury size (Figure 2), previous studies and our own preliminary data (unpublished) suggests the magnitude of the neutrophil response is at least in part dependent on sterility of the wound (Miskolci et al., 2019). This likely explains the limited neutrophil response in our laser injury model, which is largely sterile in comparison with the more external tail transection. This is an important consideration for groups attempting to model sterile injury using invasive procedures (Tsarouchas et al., 2018).

Our data show that, following laser heart injury, the initial response is dominated by neutrophils and macrophages recruited locally from the pericardial region. This contrasts with the later response where these locally recruited cells are joined by neutrophils and macrophages from more distal sites in the trunk and, in the case of macrophages, from the head. It is possible a fraction of macrophages migrating from the head to the heart injury are microglia as is the case in larval spinal cord injury, albeit a neural tissue (Tsarouchas et al., 2018). Similar examples of long-range migration have been reported in tail transection studies, where some cells are known to originate from the CHT (Yoo and Huttenlocher, 2011). However, our study is the first to comprehensively determine the ontogeny of recruited larval immune cells, which is widely appreciated to influence repair and regeneration in other models (Ginhoux et al., 2016).

The method of inflammation resolution in the regenerative larva is highly relevant to understanding conditions permissive to tissue regeneration. In our heart injury model, we found no evidence of neutrophil cell death in live imaging, despite ourselves and others reporting this occasionally occurring following tail transection (Supplementary Video 4 and Loynes et al., 2010; Hoodless et al., 2016). Our study suggests that the primary mode of resolution for both cell types in both injury models is reverse migration, previously only reported for neutrophils (Mathias et al., 2006). Following heart injury, neutrophils and macrophages dispersed randomly rather than gravitating to particular tissues. This finding is in agreement with others who have used mathematical models to demonstrate that random diffusion best describes neutrophil reverse migration (Holmes et al., 2012a,b).

In addition to determining the origin of recruited innate immune cells, we wanted to define their route of migration through the larvae. Mammals have circulating neutrophils and monocytes, supplied by the bone marrow, which can rapidly extravasate into injured tissues (Anderson and Anderson, 1976). Similarly, adult zebrafish have circulating hematopoietic lineage cells, albeit in low numbers and of kidney marrow origin (Willett et al., 1999). Our analysis of larvae in steady state confirmed that larvae usually have no circulating neutrophils or monocytes. However, we saw that in both injury models, neutrophils were indeed mobilized into the blood, but macrophages only did so following tail transection. We suspect that this difference in detection might reflect a difference in the magnitude of inflammatory response between the two injuries, as there were few circulating macrophages even in the larger response of the tail transection. Our timelapse videos of tissue macrophages entering the blood from the CHT demonstrates that it does occur, if rarely, and that perivascular crawling is the more common method of migration.

Notably, for all analyses comparing the two injury models, the laser heart injury seemed to be a milder version of the tail transection, possibly a direct consequence of injury size or increased bacterial load at the tail wound. For example, in addition to the number of neutrophils at the heart lesion being lower than that at the tail wound, fewer neutrophils are seen in circulation and no appreciable drop in CHT numbers was detectable. Nevertheless, our whole larva live imaging does suggest that the CHT is indeed the source of neutrophils suggesting that too few leave the CHT to cause a detectable reduction.

We hypothesized that neutrophils and macrophages might use additional routes and modes of migration to wounds other than circulating peripheral blood, and so performed whole larvae timelapse imaging. Our timelapse videos revealed that the majority of immune cells crawl along the outside of vessels. Interestingly, all vessel types were utilized, even developing lymphatics which were not yet fully patent. This live imaging directly confirmed the CHT as a major source of mobilized neutrophils and macrophages in both laser heart injury and tail transection. Others have reported the use of blood vessels (Manley et al., 2020), however our timelapse videos show this to be the *primary* mode of migration. We speculate that the immune adherence proteins found on endothelium might make these routes more tractable to migrating neutrophils and macrophages (von Andrian et al., 1993). Indeed, this could be an important mode of migration in larger adult wounds whose local scale is similar to that of a whole larva. Immune cells are often found adjacent to blood vessels in skin wounds, and assumed to be extravasating, but this could instead represent local migration. Additionally, we confirmed neutrophil migration to heart injury is Cxcr1/2 dependent and showed for the first time that recruitment of distal macrophages could also be Cxcr1/2 dependent. Interestingly a recent RNAseq analysis of larval zebrafish macrophages and neutrophils found Cxcr1 to only be expressed in the latter and Cxcr2 in neither (Rougeot et al., 2019). Thus suggesting that the decrease in macrophage numbers is likely an indirect, effect perhaps related to the reduced neutrophil presence at the lesion. We speculate that this may not have been observed in tail transection studies as this larger, non-sterile wound may have a greater array of compensatory chemoattractant signals than our sterile heart laser injury lesion. Antagonism of established chemokine axes such as CCL8-CXCR1/2 modulates local immune cell trafficking and might have implications for repair and revascularisation attempts in the human heart following myocardial infarction.

One of the key purposes of establishing a larval zebrafish model of myocardial infarction is to facilitate *in vivo* imaging live on the beating heart, as this is currently impossible using other models and other imaging modalities. The combination of heartbeat-synchronized LSFM imaging, injection of fluorescent dextran and use of a pan-nuclear reporter transgenic gave us unprecedented awareness of the local cardiac architecture in 3D timelapse. Despite the absence of supporting coronaries or lymphatics, we observed neutrophils and macrophages migrating onto the injured heart via adjacent pericardium and settling precisely over the lesion. The high temporal and spatial resolution facilitated characterization of immune cell behavior at the injured myocardium, where neutrophils and macrophages become more meandering and migrate further. Neutrophils and macrophages were not seen to interact with each other, thus excluding efferocytosis of neutrophils by macrophages as a form of inflammation resolution in this model.

Imaging neutrophils and macrophages simultaneously using timelapse LSFM led to the unexpected discovery that a small population of motile immune cells are marked by both the neutrophil marker *mpx* and macrophage marker *mpeg1*. Although, *mpeg1* is known to mark B-cells in the adult (Ferrero et al., 2020), in larvae these markers are considered mutually exclusive and a pan macrophage marker (Ellett et al., 2011). To our knowledge the only other evidence of such cells comes from the previously mentioned bulk RNAseq study characterizing larval zebrafish macrophage and neutrophil gene expression (Rougeot et al., 2019). The RNAseq showed low levels of *mpx* expression in *mpeg1*+ cells which the authors reasonably interpreted as general overlap in gene expression between macrophages and neutrophils. The cellular level resolution of our study allows us to suggest that this low expression of *mpx* in *mpeg1*+ cells can be explained by the contribution of a small number of *mpx*+ *mpeg*+ neutrophils. We are confident that these cells are indeed neutrophils because their size, shape, chemokine requirements and behavior were all distinguishable from macrophages, but indistinguishable from neutrophils.

Furthermore, we were able to provide evidence against the most likely alternative explanations for co-positivity. Subcellular analysis of fluorophore distribution suggested that the *mpeg1*:mCherry signal was from endogenous expression rather than acquired by phagocytosis of macrophage material. Another possible explanation is that neutrophil *mpeg1*:mCherry expression delineates unactivated neutrophils and activated neutrophils. This seems unlikely as both types of neutrophil are found quiescent in the CHT, and both are found actively participating in the wound inflammatory environment. Additionally, our flow cytometry data indicated both types undergo degranulation upon wounding, suggesting neither represent a cell that was already activated. However, the fact that there is underlying transcriptional heterogeneity leading to differential expression of this gene, is contrary to the idea that neutrophils are all homogenous. Similarly this deduction holds true irrespective of whether co+ cells possess true *mpx mpeg1* co-expression or if it is unfaithful transgene expression. The fact remains that co-positivity only occurs in a subset of neutrophils suggesting an underlying heterogeneity in transcriptional regulation within larval zebrafish neutrophils. Furthermore, *mpeg1* has a specific immune function, as it encodes a pore protein for microbial defense (Benard et al., 2015) and hence these cells might provide enhanced anti-microbial activity in non-sterile wounds. The co-positive cells are an intriguing finding that might have been previously overlooked as *mpeg1*:mCherry and *mpx*:GFP transgenes are often not imaged simultaneously. Further investigation, ideally by single cell RNAseq, is required to understand how these cells might differ in function from single-positive neutrophils and to test for further innate immune cell heterogeneity.

In summary, we have validated a larval zebrafish model of heart injury suitable for the study of immune cell migration. Our work proposes a larval immune cell multi-stage recruitment model (Figure 8) whereby macrophages and neutrophils egress from the CHT to travel via the blood or vasculature to the injury site. Local immune cells from the initial wave of cells, are later joined by neutrophils and macrophages from more distal sites. Both macrophage and neutrophil numbers then resolve by reverse migration, randomly dispersing into adjacent tissues. We also highlight the presence of a neutrophil subtype defined by its expression of the macrophage marker *mpeg1*. Future studies will be required to understand the role of these immune cells in the injured larval zebrafish heart and whether they can be modulated to improve cardiac regeneration.

## Materials and Methods

### Zebrafish Husbandry and Strains Used

Zebrafish husbandry, embryo collection and maintenance were conducted as per standard operating procedures. This was in accordance with the Animals (Scientific Procedures) Act, 1986 and approved by The University of Edinburgh Animal Welfare and Ethical Review Board in a United Kingdom Home Office-approved establishment. All experiments were performed on staged animals aged between 72–120 hpf (Kimmel et al., 1995). The following transgenic zebrafish were used: *Tg(myl7:eGFP)*^*twu*26^ (Huang et al., 2003), *Tg(myl7:h2b-GFP)*^*zf*52^ (Mickoleit et al., 2014), *Tg(myl7:DsRed2-NLS)*^*f*2^ (Rottbauer et al., 2002), *Tg(nfkb:eGFP)*^*i*235^ (Feng et al., 2012), *Tg(mpx:mCherry)*^*uwm*7^ (Yoo et al., 2010), *Tg(mpx:GFP)*^*i*114^ (Renshaw et al., 2006), *Tg(mpeg1:mCherry)*^*gl*23^ (Ellett et al., 2011), *Tg(mpx:gal4;UAS:Kaede)*^*i*222^ (Ellett et al., 2015), *Tg(fms:Gal4.VP16)*^*i*186^, referred to as *csf1r:gal4* (Gray et al., 2011), *Tg(UAS-E1b:NfsB-mCherry)*^*c*264^, abbreviated to *UAS:mCherry-NTR* (Davison et al., 2007), *Tg(fli1:eGFP)*^*y*1*tg*^ (Lawson and Weinstein, 2002), *Tg(kdrl:mCherry)*^*ci*5^ (Proulx et al., 2010), *Tg(kdrl:GFP)*^*la*116^ (Choi et al., 2007), and *Tg(h2a:GFP)* (Pauls et al., 2001). Adults were day-crossed as appropriate to yield desired combinations of transgenes in embryos. Embryos were treated with 0.003% phenylthiourea (Fisher Scientific, Hampshire, New Hampshire) at 7 hpf to prevent pigment formation and enhance image clarity (Karlsson et al., 2001). Fish were housed at 28.5°C in conditioned media and imaged at room temperature (23°C) on various microscopes (details below). When necessary, larvae were periodically anesthetized using 40 μg/ml tricaine methanesulfonate (Sigma Aldrich, St Louis, Missouri) before being transferred back to conditioned media.

### Localized Ventricular Laser Ablation

A Zeiss Photo Activated Laser Microdissection (PALM) laser system (Zeiss, Oberkochen, Germany) (Supplementary Figure 1-supplement 1) was used to induce a localized injury at the ventricular apex of anesthetized 72 hpf larvae as initially reported recently (Taylor et al., 2019). Larvae were laterally mounted on a glass slide in 20 μl anesthetized conditioned media and the laser was fired through a 20X objective. Injuries were deemed successful when ventricular contractility noticeably decreased, the apex had shrunk, and the myocardial wall had swollen without causing cardiac rupture and subsequent erythrocyte leaking (Figure 1B). A successful injury resulted in the segment of dysfunctional tissue losing fluorescent myocardial transgenic signal (Figure 1D). Uninjured (control) larvae were treated in the same manner up to the point of laser injury, when they were individually separated into single wells of a 24-well plate and maintained in the same environmental conditions as injured fish.

### Tail Fin Transection

The tail fin of anesthetized 72 hpf larvae was transected using a sterile scalpel, avoiding damage to the end of the notochord and vasculature (as depicted in the Figure 2 schematic), and as previously reported (Hoodless et al., 2016). Uninjured (control) fish were treated in the same manner up to the point of transection, when they were separated into a 24-well plate and maintained in the same environmental conditions as injured fish.

### Epifluorescence Microscopy

A Leica M205 FA stereomicroscope (Leica, Wetzlar, Germany) with GFP, mCherry and BFP filters was used for all serial timepoint epifluorescence imaging experiments. To visualize neutrophil or macrophage presence at heart or tail wounds, larvae were anesthetized and mounted laterally on a glass slide in 50 μl of conditioned media. The number of immune cells on the heart were quantified by manually observing and counting cells moving synchronously with the beating heart. Heart images were acquired using 16X objective. The number of immune cells at the tail were quantified by counting from the caudal end of the vascular loop to the wound edge as performed by others (Miskolci et al., 2019). Tail images were acquired using 8X objective. CHT images were acquired using a 6X objective. Whole larvae images were acquired using 2.5X objective. The number of circulating immune cells were quantified by manually observing whole larvae for liberated cells which can be detected in real time migrating through or around the vasculature.

An EVOS Auto2 system (ThermoFisher, Waltham, MA, United States) with GFP, RFP and DAPI filters was used to image whole larvae in epifluorescence timelapse, in an automated manner at 28.5°C. Larvae were anesthetized and mounted laterally in a 6-well plate in 1% agarose. Three larvae were mounted per well. To ensure larvae remain immobilized, 500 μl of anesthetic solution was added to each well. Images of each well were acquired using a 2X objective at 1-minute intervals up to 24 hpi.

### Light Sheet Fluorescence Microscopy (LSFM)

Individual larvae were prepared for LSFM by embedding in 1% low melting-point agarose (ThermoFisher, Waltham, Massachusetts) in anesthetized conditioned media inside FEP tubes (Adtech Polymer Engineering, Stroud, United Kingdom). This embedding reduced gradual drift of the embryo in the FEP tube, without causing developmental perturbations during long-term imaging. Larvae were used only once for a timelapse imaging experiment, and any repeats shown come from distinct individuals. Larvae were mounted head down such that the heart faces toward both illumination and imaging objectives to improve image clarity. All LSFM experiments were performed at room temperature (23°C). The setup of our custom-built LSFM system has been previously reported in detail (Taylor et al., 2019).

### Optically Gated (Beat-Synchronized) Heart Imaging

Real time prospective optical gating and more recent adaptive prospective gating methods in conjunction with LSFM have been previously published (Taylor et al., 2011, 2012, 2019). In summary, the software permits real-time 3D imaging of the normally beating heart by determining the phase of the cardiac cycle using images acquired from a separate bright-field camera. The software checks subsequently acquired bright-field images against this reference image-set in real-time and activates the imaging laser at a user-designated phase of the cardiac cycle. This ensures the lightsheet only strikes the heart when it is in that desired phase of the cardiac cycle, illuminating each z-plane for a few milliseconds and limiting the impact of phototoxicity and photobleaching. Synchronization is maintained over tens of hours by periodically updating the reference images to account for changes in appearance of the heart during development/injury (Taylor et al., 2019). For timelapse imaging, the entire heart is scanned in 3D every 3 mins, with a z-plane spacing of 1 μm, and each image stack is stored as a.tif file. These images can be viewed as a maximum intensity projection images during acquisition in order to, if necessary, optimize the quality of subsequently acquired images.

### Histological Cell Death Assays

To detect cell death at the injured ventricle, whole-mount larval TUNEL staining was performed. Larvae were fixed in 4% PFA for 30 mins and transferred to 1:10 dilution of PBS. Larvae were subsequently digested in 1 μg/ml Proteinase K for 1 h. Larvae were re-fixed in 4% PFA for 20 mins and subsequently washed in PBT. TUNEL staining was performed using ApopTag Red *In situ* kit (MilliporeSigma, Burlington, MA, United States) to label apoptotic cells, as described in (Buckley et al., 2017). Stained hearts were imaged using LSFM.

To corroborate these findings acridine orange staining was performed. Larvae were incubated in 10 ng/μl of Acridine orange solution (ThermoFisher, Waltham, MA, United States) in the dark for 20 min. Larvae were subsequently washed three times in conditioned media for 10 min and imaged using heartbeat synchronized LSFM.

For live staining with propidium iodide, larvae were injected intravenously with a 1 nl volume of 1 mg/ml PI (ThermoFisher, Waltham, MA, United States) in PBS immediately following laser injury (∼15 minutes post injury) and imaged using heartbeat synchronized LSFM.

### Photoconversion of Neutrophils and Macrophages

We established a specific protocol for photoconversion of neutrophils and macrophages following laser heart injury, using *Tg(mpx:gal4;UAS:kaede)* and *Tg(csf1r:gal4;UAS:kaede)* larvae respectively on a Leica M205 FA epifluorescence stereomicroscope (Leica, Wetzlar, Germany). To increase throughput, we generated a digital reticule informing us of the beam location on the larva. The reticule was specifically positioned to target one of two different regions; the head and/or pericardium. Larvae were mounted laterally in 50 μl of conditioned media on a glass slide prior to photoconversion. The beam was focused through a Leica PLAN APO 2.0x CORR Objective with an eye piece magnification of x16. A Mercury Vapor Lamp (Leica, Wetzlar, Germany) at 50 W power was used in conjunction with a BFP filter to photoconvert kaede-labeled immune cells. Depending on the experiment, larvae were either differentially photoconverted at 1 h prior to heart injury and imaged at 2 and 6 hpi (immune cell tissue origin assay), or their pericardiums were fully photoconverted at 6 hpi and imaged at 24 hpi (immune reverse migration assay). For the immune cell tissue origin experiment, the head was first fully photoconverted from green to red by exposing larvae for 60 s (until no green kaede signal was detectable above background). Next, the pericardium was semi-converted from green to brown (red and green) by exposing larvae for 30 s (immune cells retain 1:1 ratio of unconverted vs converted kaede fluorophore). The remainder of the larvae (which we term trunk) maintained endogenous green kaede fluorophore, hence producing three differentially labeled immune cell populations in the same larvae. Larvae were not imaged beyond 24 hpi because, shortly after this elapsed time, converted kaede fluorophore became difficult to detect in semi-converted cells. For the immune cell reverse migration experiment, the pericardium was fully photoconverted from green to red by exposing larvae for 60 s (until no green kaede signal was detectable above background). We confirmed that 93 ± 2.7% of pericardial immune cells photoconverted at 6 hpi were able to be traced at 24 hpi following their reverse migration, therefore validating the sensitivity of this technique (Supplementary Figure 3-supplement 1B). Larvae were imaged using both GFP and mCherry filters to detect level of photoconversion.

### Pharmacological Cxcr1/2 Inhibition

Larvae were preincubated in 5 μM SB225002 (Sigma Aldrich, St. Louis, MO, United States) or 0.1% DMSO vehicle (Sigma Aldrich, St. Louis, MO, United States) dissolved in conditioned media at 70 hpf for two hours prior to heart or tail injury. Following injury, larvae were continuously bathed in drug or vehicle throughout the duration of the experiment.

### Fluorescent Dextran Injection

Custom-synthesized molecular weight (500 kDa) pacific blue fluorophore-labeled dextran (Fina Biosolutions, Rockville, MD, United States) was injected into larvae at 1 hpi. Injections were administered in a 1 nL volume of 5% (w/v) dextran dissolved in phosphate-buffered saline and injected into the cardiac sinus venosus as described in more detail previously (Rider et al., 2018).

### Image Analysis

Unless otherwise stated, images were prepared, processed and analyzed using ImageJ (Fiji) software (National Institutes of Health, Bethesda).

### Ventricular Ejection Function

*Tg(myl7:GFP)* hearts were imaged using a Leica M205 FA epifluorescence stereomicroscope (Leica, Wetzlar, Germany) to capture when the ventricle was in diastole and systole. The ventricular area in diastole and systole was measured and ventricular ejection fraction calculated using the formula 100 X [(Diastolic Area – Systolic Area)/Diastolic Area] (Matrone et al., 2013).

### Photoconverted Immune Cells

Epifluorescence images of photoconverted larvae were analyzed to quantify the number of immune cells that were fully converted (red), semi-converted (red and green) or unconverted (green) on the heart or to identify immune cell locations in the whole larvae following heart injury. More specifically, for the latter reverse migration (immune cell location) assay, each larva was divided into 48 equally sized square zones (3 rows of 16, individual zone size = 31000 μm^2^). The number of photoconverted immune cells within each square zone at 24 hpi was quantified per larvae, averaged and normalized per zone to generate a heat map (Figure 3D).

### Immune Cell CHT Quantity

The number of immune cells specifically within the CHT (as depicted in Figure 4) were quantified across timepoints and normalized to baseline preinjury (0 hpi) for each larva. Quantification was performed in a semi-automated fashion using a custom FIJI. In brief, a rectangular region of interest (766 μm × 151 μm) is generated and positioned over the CHT. This region is cropped, and brightness/contrast enhanced to identify all neutrophils or macrophages. Background signal is subtracted, then an unsharp mask and watershed segmentation are applied. The ‘Analyze Particles’ plugin is run to measure the area of segmented neutrophils or macrophages in the CHT.

### EVOS Whole Larva Imaging Analysis

Timelapse images were analyzed using the FIJI plugin Trackmate (Tinevez et al., 2017) automating the tracking of neutrophils and macrophages. Detection parameters: LOG detector, diameter 14 μm and threshold 5 μm. Tracking was performed using Simple Linear Assignment Problem (LAP) algorithms with parameters: linking distance 27 μm, max closing distance 15 μm and gap closing max frame gap 2. The dataset was then handled one of two ways depending on the analysis.

Analysis 1 - vessel use incidence: the incidence of use of each vessel for lasered datasets was obtained manually by plotting a line along the dorso-ventral axis one somite anterior to the cloaca and counting how many cells pass the line over the time frame 1–12 hpi for each vessel. Detecting and ringing each cell using Trackmate aids counting. The same method was used for tail transection datasets except the dorso-ventral line was drawn four somites back from the tail fin. The reasoning behind using different sites for quantification is that the majority of migrating cells will be coming from the CHT, which is located between the two different wound sites we studied, and hence cells are expected to move in opposite directions depending on the injury model.

Analysis 2 - cell behavior: tracked cells were filtered to a range of 1.5–8 hpi and restricted to the trunk region of the larva. Non-migrating cells were then excluded from the analysis based on displacement. Remaining tracks were used for plots of speed and meandering index. Meandering index was calculated using the Trackmate output data ‘Displacement’, ‘Duration’ and ‘Speed.’ Where Meandering index = Displacement/(Duration/Speed).

### Surface Rendering of Heart, Vasculature, Nuclei and Tracking of Immune Cells Following Heart Injury

Light sheet fluorescence microscopy *z*-stacks of the myocardium, endocardium, immune cells and nuclei were surface-rendered using Imaris software (Bitplane, Zurich, Switzerland). Rendered immune cells were tracked during the indicated time course using an autoregressive motion algorithm, and individual tracks were surface rendered for display using Imaris.

### Differentially Color-Labeling Cell Types Imaged in the Same Fluorescent Channel

To distinguish *kdrl:mCherry* fluorescence from *mpeg1:mCherry* fluorescence, a sequential subtraction of two frames difference was performed across the image sequence to produce a magenta channel where only moving cells were visible. This distinguished moving macrophages (magenta) from the static endothelium (blue). To distinguish *mpx:GFP* fluorescence from *h2a:GFP* fluorescence, a threshold was applied across the image sequence, creating a mask for brighter *mpx:GFP* neutrophils only. The brightness/contrast of *h2a:GFP* fluorescence (gray) was altered so that it was visible when overlaid with the *mpx:GFP* fluorescent mask (green). All channels were combined to create a composite image displayed in Figure 6D.

### Ventricle-Recruited Immune Cell Behavior Analysis

Light sheet fluorescence microscopy timelapses of the myocardium and neutrophils or macrophages were processed as maximum intensity projections. The Fiji plugin MTrackJ (Meijering, 2006) was used to manually track the migration of immune cells from the moment they migrate onto the ventricular myocardium until the moment they reverse-migrate off the ventricular myocardium or, as is commonly the case for macrophages, until the end of the timelapse.

### Immune Cell Behavior Analysis Following Tail Transection Using LSFM Timelapses

LSFM timelapses of the transected tail fin acquired at scan intervals of 1 min were analyzed using Imaris (Bitplane, Zurich, Switzerland). Default and suggested parameters were used for supervised tracking by the autoregressive motion algorithm. Co-positive cells were designated as cells with a mpx:mpeg1 ratio less than 3:1 and greater than 1.33:1. Gating the cells by ratios overcomes the variability in absolute intensity as cells move through different area of tissue and take on different shapes. Plotted values are calculated from the averages of all cells in a particular larva 2–3 hpi following tail transection.

### Fluorescence Intensity Plots of Immune Cell *z*-Planes

LSFM z-stacks of transected tail fins were analyzed in Fiji using the plot profile function to measure the intensity of the desired fluorophore across a manually drawn line.

### Flow Cytometry

Larvae were euthanized using 0.3 mg/ml tricaine methanesulfonate at 24 h post injury (4 dpf) and transferred into 1 ml of enzyme digestion mix consisting of Collagenase V (Sigma Aldrich, St. Louis, MO, United States), Collagenase D (Roche, Basel, Switzerland), Dispase (ThermoFisher, Waltham, MA, United States) and DNase (Roche, Basel, Switzerland). A single-cell suspension in RPMI+ 10% FBS was achieved through mechanical homogenization and filtering through a 40 μm cell strainer. The cells were resuspended in HBSS (−/−)+ 15 mM HEPES+ 25 μM D-Glucose) and filtered through a 40 μm cell strainer. Lastly, DAPI (1:1000) was added to the sample which was subsequently analyzed by flow cytometry. Samples were kept on ice throughout and were analyzed by flow cytometry at 4°C. Analysis and visualization of data was performed using FCS Express 7 software (*De Novo* Software, Pasadena, CA, United States).

### Statistical Analysis

Graphs and statistics were curated in GraphPad Prism 8 software (GraphPad Software, San Diago, CA, United States). Data were analyzed by student *t*-test, one-way ANOVA or two-way ANOVA followed by an appropriate multiple comparison *post hoc* test. All statistical tests, *p*-values and *n* numbers used are given in figure legends.

## Data Availability Statement

All datasets presented in this study are included in the article/Supplementary Material.

## Ethics Statement

The animal study was reviewed and approved by The University of Edinburgh Animal Welfare and Ethical Review Board.

## Author Contributions

AK, FAB, and MAD conceived and designed the study. AK, FAB, MEMO, and CB carried out all experiments. Image analysis was performed by AK, FAB, and CB. LSFM-related technical assistance was provided by JMT. AK and FAB wrote the manuscript. CB, JMT, CST, JJM, AGR, and MAD edited the manuscript. MAD, AGR, and CST supervised the study. All authors contributed to the article and approved the submitted version.

## Conflict of Interest

The authors declare that the research was conducted in the absence of any commercial or financial relationships that could be construed as a potential conflict of interest.
