# Supplementary material for: Live Imaging of Heart Injury in Larval Zebrafish Reveals a Multi-Stage Model of Neutrophil and Macrophage Migration
Source: Front Cell Dev Biol. 2020 Oct 19;8:579943. doi: 10.3389/fcell.2020.579943 (PMC7604347; doi:10.3389/fcell.2020.579943)
Supplement: Supplementary file 14 [file Data_Sheet_1.docx]

Supplementary Material


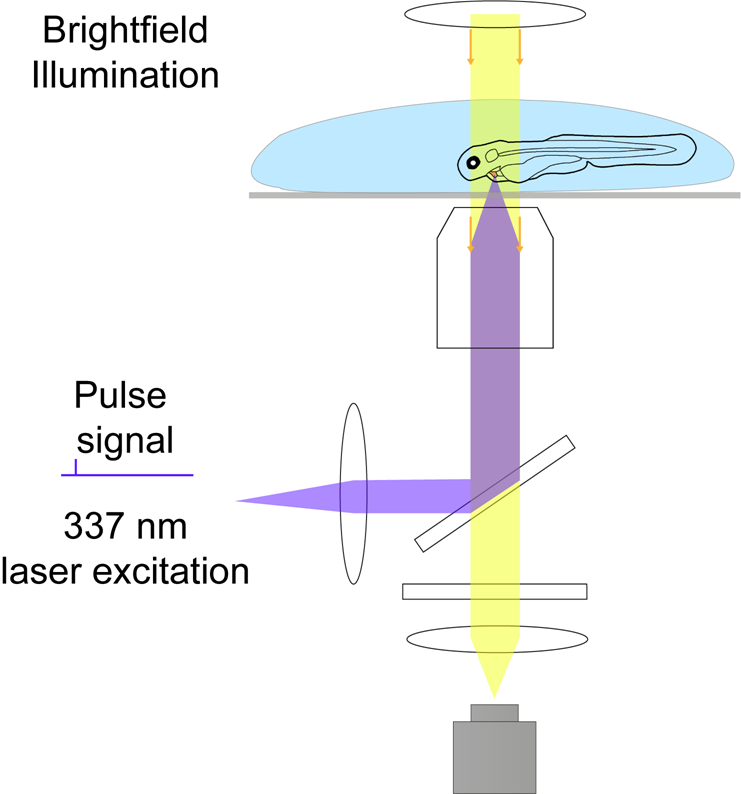


**Figure 1 – supplement 1**

**Summary of targeted heart laser injury using the Zeiss PALM laser system**

Larvae are mounted laterally in anaesthetised conditioned medium on a glass slide and brought into a central view using the transmission brightfield illumination (yellow). A tightly focused laser pulse is brought in through the back port of the microscope body (purple) and focused onto the sample using a 20X objective. Pulses are ~3ns duration at 337nm and are initiated by the user through the software. Only images from the brightfield illumination are acquired by the camera.


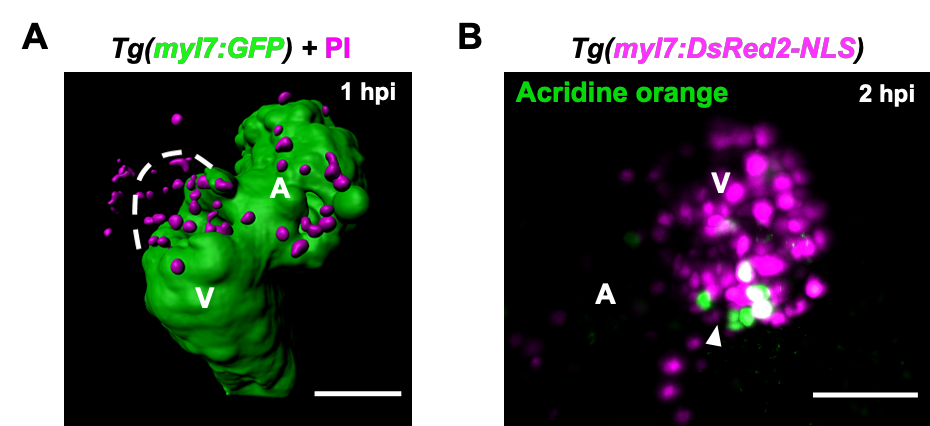


**Figure 1 – supplement 2**

**Propidium iodide and acridine orange staining demonstrate necrotic and apoptotic cells at the heart lesion respectively**

(A) 3D render of a LSFM z-stack from a 3dpf *Tg(myl7:GFP)* larva injected with propidium iodide following injury (1 hpi) to stain nuclei of necrotic cells (magenta). Loss of myocardial GFP is indicated with a dashed line. Scale bar = 100μm. (B) 3D LSFM image of an acridine orange stained (green) *Tg(myl7:DsRed2-NLS)* injured heart at 2 hpi displaying cardiomyocyte nuclei (magenta). Injury site is marked with a white arrowhead that is surrounded by apoptotic (acridine orange stained) cardiomyocytes. Image displayed as a MIP. Scale bar = 50μm. V, ventricle; A, atrium.


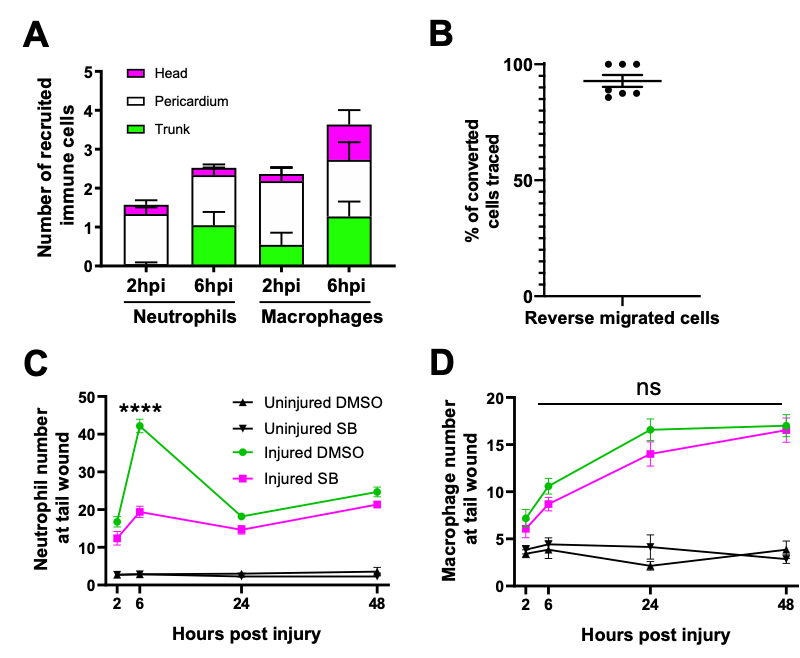


**Figure 3 – supplement 1**

**CXCR1/2 antagonist SB225002 inhibits neutrophil but not macrophage migration to tail transection**

(A) Number of neutrophils and macrophages recruited to the injured ventricle at 2 hpi and 6 hpi according to tissue origin (head, pericardium or trunk), n = 11-21 larvae, experimental n = 3. (B) Percentage of pericardial neutrophils photoconverted at 6 hpi that were able to be traced at 24 hpi following reverse migration thus validating the sensitivity of the technique, n = 7 larvae, experimental n = 3. Number of neutrophils (C) and macrophages (D) at the tail transection wound of *Tg(mpx:GFP;mpeg1:mCherry)* larvae treated with pharmacological CXCR1/2 antagonist SB225002 (5μM) or DMSO (0.1%) vehicle, n = 7-14 larvae, experimental n = 3. Two-way ANOVA and Tukey post hoc test performed for immune cell comparisons between injured treatment groups where ***** p<0.0001*; ns, non-significant. Error bars = SEM for all graphs.


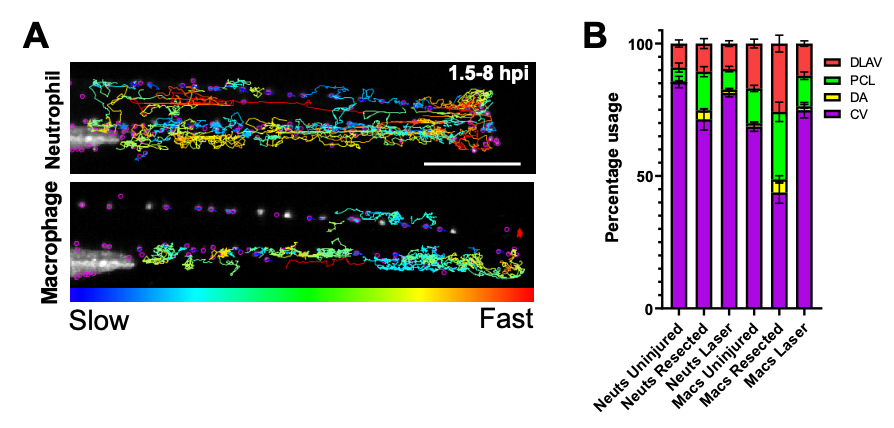


**Figure 5 – supplement 1**

**Macrophages and neutrophils utilise PCLs following injury for fast migration**

(A) Neutrophil and macrophage tracks colour-coded by speed showing PCL use by neutrophils for fast migration following tail transection (1.5-8 hpi). Tracks were used to generate speed and meandering data in Figure 5G and 5H. Scale bar = 500μm. (B) Percentage usage of trunk vessels by neutrophils and macrophages following tail transection and heart laser injury, derived from the same timelapse datasets as Figure 5D and 5E. Error bars = SEM, n = 4-6 larvae analysed per group. DLAV = dorsal lateral anastomotic vessel, PCL = parachordal lymphatic, DA = dorsal aorta and CV = cardinal vein.


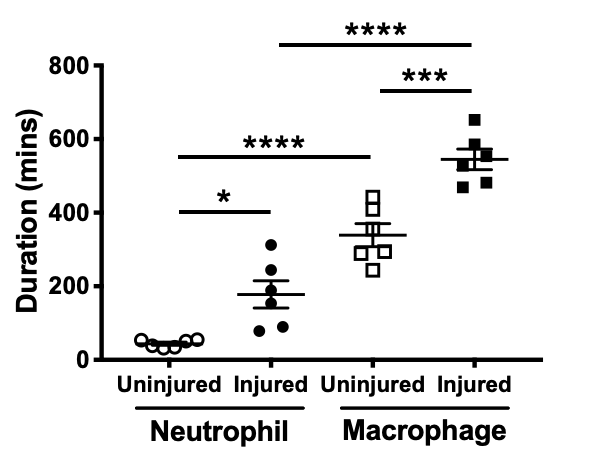


**Figure 6 – supplement 1**

**Following recruitment macrophages migrate on the ventricle for longer periods of time compared to neutrophils during steady state and following injury**

Duration of neutrophil and macrophage migration on the ventricle following heart injury and in uninjured larvae. Average cell behaviours are plotted per larva, n = 5 cells tracked per larva and n = 6 larvae per group. Error bars = SEM. One-way ANOVA and Tukey post hoc test performed between mean behavioural values for each larva, where * *p<0.05*, *** *p<0.001* and **** *p<0.0001*.


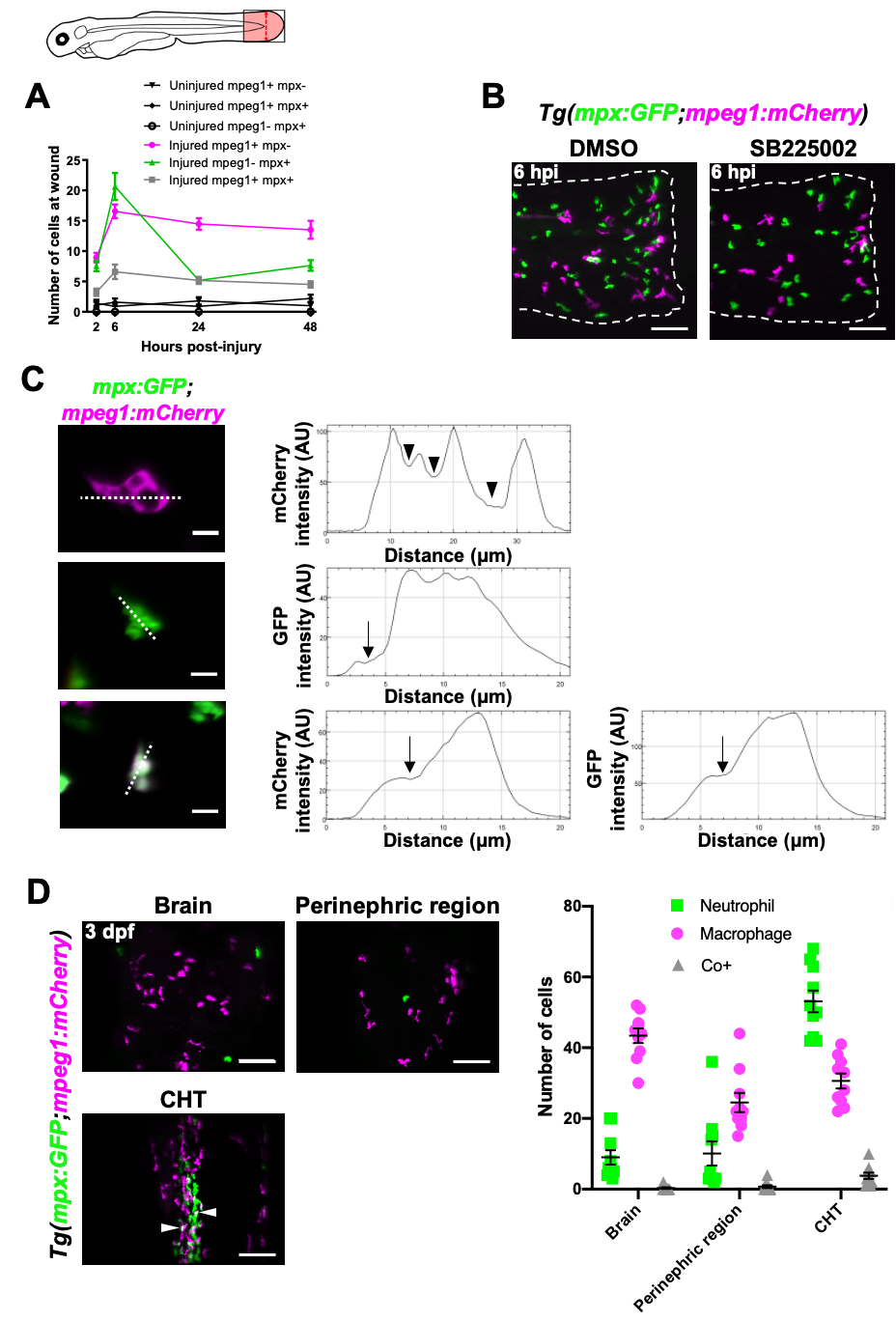


**Figure 7 – supplement 1**

**Co-positive cells are not engulfed macrophages or immature neutrophils**

(A) Number of macrophages, neutrophils and co-positive cells at the tail transection wound across the indicated timepoints. Error bars = SEM, n = 10-24, experimental n = 3. (B) Epifluorescence images of transected tail fins from *Tg(mpx:GFP;mpeg1:mCherry)* larvae at 6 hpi, subsequent to bathing in CXCR1/2 antagonist SB225002 (5μM) or DMSO (0.1%) vehicle. Scale bar = 100μm. (C) LSFM z-plane image from the wound of a tail transected *Tg(mpx:GFP;mpeg1:mCherry)* larva, showing a macrophage (top), neutrophil (middle) and co-positive cell (bottom). Fluorescence intensity plots derived from lines drawn across each cell demonstrating the extent of signal uniformity. Black arrowheads mark signal troughs caused by phagosomes and black arrows mark signal troughs caused by nuclei. Scale bar = 10μm. n = 6 cells analysed. (D) LSFM 3D images from specified regions of *Tg(mpx:GFP;mpeg1:mCherry)* larvae showing the mixture of neutrophils, macrophages and co-positive cells (labelled with white arrowheads) at each indicated region (left). Image displayed as MIPs. Number of neutrophils, macrophages and co-positive cells in the three specified regions (Brain, Perinephric region and CHT) (right). Error bars = SEM, n = 10 larvae analysed per group.


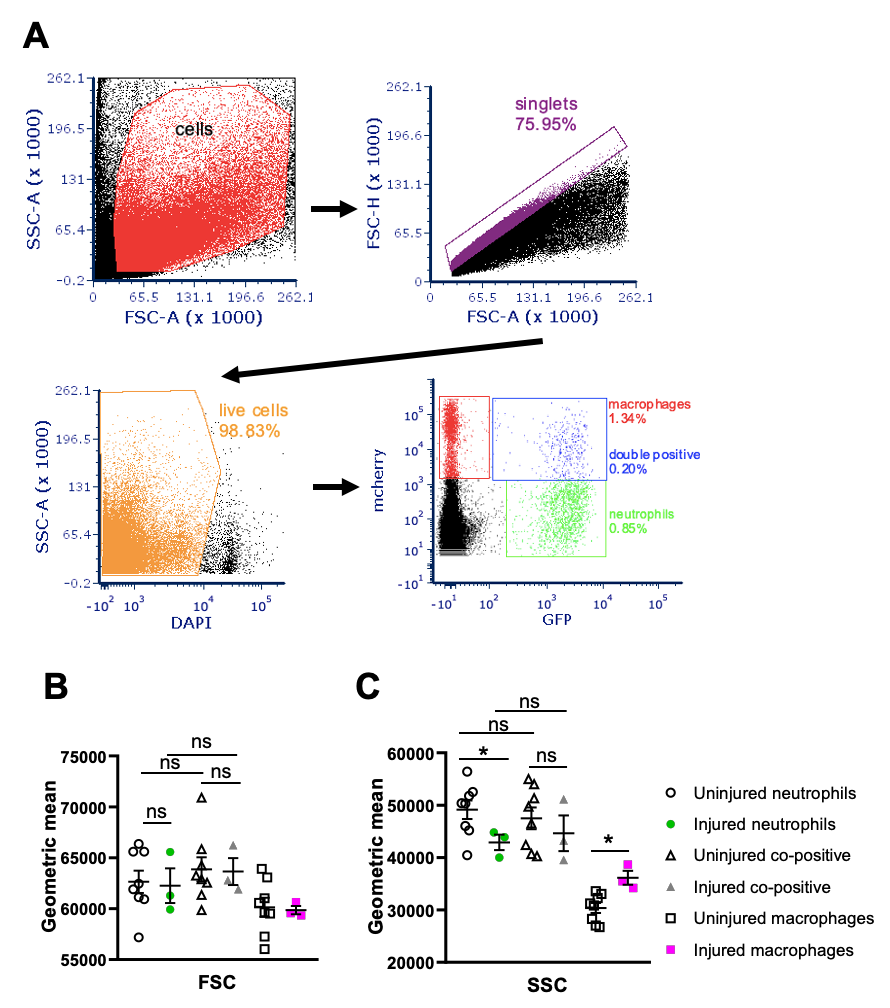


**Figure 7 – supplement 2**

**Flow cytometry gating strategy**

(A) Plots describing the gating strategy for the FACS recovery of macrophages and neutrophils from dissociated 4 dpf *Tg(mpx:GFP;mpeg1:mCherry)* zebrafish larvae. A combination of forward scatter (FSC), side scatter (SSC), DAPI, and endogenous transgene fluorescence was used to isolate pure populations of live neutrophils, macrophages and co-positive cells, as indicated. Mean FSC (B) and SSC (C) for each sorted population. Comparisons between groups were performed by one-way ANOVA followed by a post hoc two-stage step-up method of Benjamini, Krieger and Yekutieli FDR correction where ** p<0.05* and ns, non-significant. Error bars = SEM for all graphs, n = 3-8 samples were analysed per group.


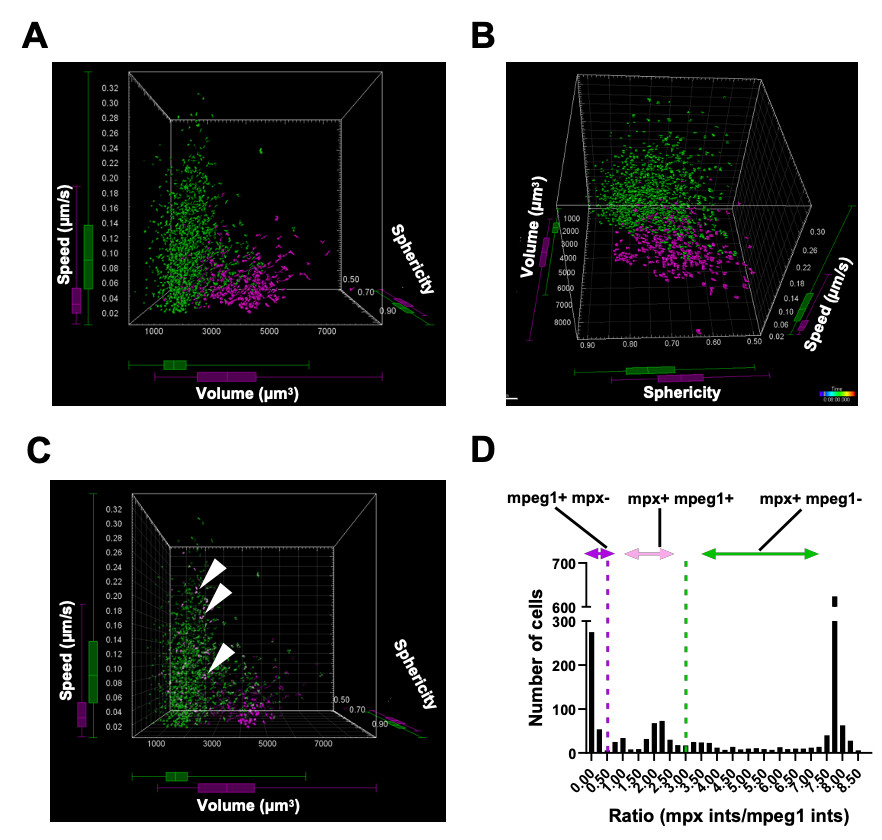


**Figure 7 – supplement 3**

**Imaris analysis of tail** **transected *Tg(mpx:GFP;mpeg1:mCherry)* larvae imaged by LSFM**

(A) 3D surface render plot of mpeg1+ and mpx+ cells indicating speed, sphericity and volume, which shows macrophages (mpeg1+, magenta) and neutrophils (mpx+, green) to segregate. (B) The same 3D plot as (A) but rotated for vantage. (C) 3D MIP plot of mpeg1+ and mpx+ cells across timepoints, indicating speed, sphericity and volume shows co-positive cells to segregate with neutrophils (white arrowheads). (D) Histogram of *mpx:GFP;mpeg1:mCherry* fluorescence intensity ratio for cells rendered in (A) showing our gating strategy for macrophages, neutrophils and co-positive cells. Lines represent boundaries where mpx:mpeg1 ratio is 3:1 or 1:3 and enclose the co-positive cell population.

**Videos (uploaded individually as additional files)**

**Dropbox storage link for alternative access to videos:**

<https://www.dropbox.com/sh/7yifviv2p5hr3dg/AABSPyh3EQ8DdVaADssxDPaqa?dl=0>

**Video captions**

**Video 1 – Uninjured and heart lasered *Tg(myl7:GFP)* heart beating in real-time**

Epifluorescence-acquired real-time video of uninjured and heart lasered (2 hpi) *Tg(myl7:GFP)* hearts. The lasered heart displays a lack of contraction and myocardial GFP loss at the ventricular apex.

**Video 2 – Neutrophil presence at the injured ventricular apex in real-time**

Epifluorescence-acquired real-time video of neutrophils following recruitment to the ventricular apex at 2hpi in a *Tg(myl7:GFP;mpx:mCherry)* larva.

**Video 3 – Neutrophils cyclically migrating around the injured heart in real time**

Epifluorescence-acquired real-time video of *Tg(mpx:mCherry)* labelled neutrophils cyclically migrating around the injured heart. Heart chambers and injury site are indicated.

**Video 4 – Neutrophil death at the tail wound edge follow transection**

LSFM timelapse of neutrophil death in a *Tg(mpx:GFP;mpeg1:mCherry)* larva following tail transection. Neutrophils about to undergo cell death are indicated. 3D images displayed as maximum intensity projections.

**Video 5 – Macrophage death at the tail wound edge follow transection**

LSFM timelapse of macrophage death in a *Tg(mpx:GFP;mpeg1:mCherry)* larva following tail transection. The macrophage about to undergo cell death is indicated. 3D images displayed as maximum intensity projections.

**Video 6 – CHT egress, rolling and free circulation of neutrophils**

Epifluorescence video of the CHT of a tail transected *Tg(mpx:GFP; mpeg1:mCherry)* larva at 6 hpi, showing neutrophil rolling and flowing in the caudal vein and neutrophil CHT egress.

**Video 7 – CHT egress and rolling of macrophages**

Epifluorescence-acquired timelapse of the CHT of a heart lasered *Tg(mpx:GFP;mpeg1:mCherry)* larva 6 hpi showing macrophage rolling in the caudal vein and macrophage CHT egress.

**Video 8 – Whole larvae timelapse of neutrophil and macrophage migration following tail transection and heart laser injury**

Epifluorescence video of three *Tg(mpx:GFP;mpeg1:mCherry)* whole larvae representing uninjured, tail transected and heart lasered neutrophil and macrophage immune responses. Larvae were injected with high molecular weight dextran to highlight blood and lymphatic vessels.

**Video 9 – Neutrophils and macrophages migrate around the pericardium prior to migrating on the heart following injury**

Surface rendering of LSFM-acquired, heartbeat-synchronised z-stack of a heart lasered *Tg(kdrl:mCherry;mpx:GFP;mpeg1:mCherry;h2a:GFP)* larva. Pericardial nuclei are labelled as small multi-coloured dots, neutrophils are labelled as large green dots, macrophages are labelled as large magenta dots and the endothelium is labelled as a red surface. Individual neutrophils and macrophages have been tracked for 30mins from 9.5 hpi and their associated migration is indicated with green and magenta tracks respectively.

**Video 10 – Neutrophil recruitment and resolution following heart laser injury**

LSFM heartbeat-synchronised timelapse of a *Tg(myl7:GFP;mpx:mCherry)* larva showing neutrophil recruitment, swarming and resolution following laser injury. 3D images displayed as maximum intensity projections.

**Video 11 – Macrophage recruitment following heart laser injury**

LSFM heartbeat-synchronised timelapse of a *Tg(myl7:GFP;mpeg1:mCherry)* larva showing macrophage recruitment following laser injury. 3D images displayed as maximum intensity projections.

**Video 12 – Neutrophil and macrophage migration following heart laser injury**

LSFM heartbeat-synchronised timelapse of a *Tg(mpx:GFP;mpeg1:mCherry)* larva showing neutrophil and macrophage migration following laser injury. 3D images displayed as maximum intensity projections.

**Video 13 – Co-positive immune cell recruitment to tail wound following transection**

LSFM timelapse of a tail transected *Tg(mpx:GFP;mpeg1:mCherry)* larva showing co-positive cells migrating to the wound. 3D images displayed as maximum intensity projections.
